# Mitochondria-targeted supramolecular coordination container encapsulated with exogenous itaconate for synergistic therapy of joint inflammation

**DOI:** 10.7150/thno.70623

**Published:** 2022-04-04

**Authors:** Xuzhuo Chen, Chang Li, Xiankun Cao, Xinlin Jia, Xinwei Chen, Zhenqiang Wang, Weifeng Xu, Fengrong Dai, Shanyong Zhang

**Affiliations:** 1 Department of Oral Surgery, Shanghai Key Laboratory of Stomatology & Shanghai Research Institute of Stomatology, National Clinical Research Center for Oral Diseases, Shanghai Ninth People's Hospital, College of Stomatology, Shanghai Jiao Tong University School of Medicine, Shanghai, 200011, China; 2 State Key Laboratory of Structural Chemistry, Fujian Institute of Research on the Structure of Matter, Chinese Academy of Sciences, Fuzhou, Fujian, 350002, China; 3 Department of Orthopedics, Shanghai Key Laboratory of Orthopedic Implant, Shanghai Ninth People's Hospital, Shanghai Jiao Tong University School of Medicine, Shanghai, 200011, China; 4 Department of Chemistry & Center for Fluorinated Functional Materials, University of South Dakota, Vermillion, South Dakota, 57069-2390, United States

**Keywords:** Coordination containers, Supermolecule, ROS scavenging, Joint inflammation, Exogenous itaconate

## Abstract

**Rationale:** Inflammatory macrophages and osteoclasts (OCs) play critical roles in joint inflammation, which feature the excessive production of reactive oxygen species (ROS), resulting in synovial inflammation and bone erosion. Scavenging ROS, especially by modulating mitochondrial metabolic activity, could be a desirable strategy for the management of inflammatory joints. This study aimed to develop a mitochondria-targeted supramolecular drug delivery system with exogenous and endogenous ROS-scavenging activities for the treatment of joint inflammation.

**Methods:** In this study, we utilized a zinc-based metal-organic supercontainer (MOSC) as a proton sponge and electron reservoir with outstanding proton binding capacity, extracellular ROS-scavenging ability, and biocompatibility to establish an efficient supramolecular nanocarrier for endo/lysosomal escape and mitochondrial targeting. 4-Octyl itaconate (4-OI), an itaconate derivative, served as the loaded guest for the construction of a synergistic therapeutic system for inflammatory macrophages and OCs.

**Results:** After the effective encapsulation of 4-OI, 4-OI@Zn-NH-pyr not only exhibited potent ROS-scavenging capacity, but also reduced ROS production by mediating mitochondrial respiration in inflammatory macrophages. Regarding its anti-inflammatory efficacy, 4-OI@Zn-NH-pyr ameliorated the inflammatory reaction by activating nuclear factor erythroid 2-related factor 2 (Nrf2), thus increasing the production of antioxidants, apart from the inhibition of NF-κB pathways. Additionally, receptor activator of nuclear factor-κB ligand (RANKL)-induced osteoclast differentiation and function was remarkably suppressed by 4-OI@Zn-NH-pyr. Consistent with *in vitro* observations, 4-OI@Zn-NH-pyr efficiently inhibited synovial inflammation and subchondral bone destruction in an acute arthritis model.

**Conclusion:** By using MOSCs that are highly reactive to ROS as drug-loaded matrices for the first time, this study provides an avenue for the management of severe joint inflammation by designing synergistic supramolecular drug-delivery systems with subcellular targeting and ROS-scavenging capacity.

## Introduction

Characterized by chronic pain, joint swelling, and functional disability, arthritis is a common inflammatory disease affecting millions of individuals worldwide 1, 2. The pathological features of arthritis include severe synovial inflammation, cartilage degeneration, and bone erosion, leading to the morphological and functional destruction of joint structures 3. The temporomandibular joint (TMJ) is a synovial joint susceptible to articular inflammation caused by osteoarthritis (OA), rheumatoid arthritis (RA), and systemic diseases 1, 4. Patients with TMJ involvement often suffer from chronic pain, abnormal sounds, and limited mouth opening, leading to undermined functions in chewing and articulating, with severely reduced quality of life 5, 6. In recent years, an increasing number of drugs have been developed for therapeutic use against arthritis, including non-steroidal anti-inflammatory drugs (NSAIDs), disease-modifying anti-rheumatic drugs (DMARDs), and biological antibodies 7, 8. However, the adverse side effects caused by high dosages and frequent administration, including gastrointestinal reactions, high risk of infection, bone loss, and hyperglycemia, in addition to high treatment costs, significantly restrict long-term administration 9. Therefore, there is an urgent need to develop novel therapeutic strategies with highly efficient drug delivery and fewer side effects for patients with arthritis.

Accumulating evidence has indicated that macrophages and osteoclasts (OCs) play critical roles in the progression of joint inflammation 10, 11. As one of the dominant cells infiltrating the arthritic synovium, inflammatory macrophages represent an early-stage hallmark of pathology, secreting abundant pro-inflammatory cytokines and enzymes, such as tumor necrosis factor-α (TNF-α), interleukin (IL)-1β, IL-6, and inducible nitric oxide synthase (iNOS), leading to the progressive destruction of articular cartilage and subchondral bone 12. Moreover, abundant inflammatory macrophages can differentiate into OCs to directly initiate the erosion of the bone matrix, apart from the indirect activation of OCs by stimuli produced by pathogenic macrophages and Th17 cells 13. Considering the vital roles of macrophages and OCs in arthritic progression, synchronized anti-inflammation and anti-bone resorption would be a promising therapeutic modality for severe joint inflammation.

The excessive production of reactive oxygen species (ROS) has been implicated in inflamed joints with synovial inflammation and bone erosion 14-16. As byproducts of oxidative phosphorylation (OXPHOS), ROS are extensively produced by inflammatory macrophages due to the enhanced level of metabolic flux in mitochondria 15. Multiple lines of evidence suggest that the increased oxidation of succinate, a metabolic intermediate in the tricarboxylic acid (TCA) cycle, drives mitochondrial ROS production in inflammatory macrophages via succinate dehydrogenase (SDH), thus upregulating the pro-inflammatory HIF-1-IL-1β axis 17, 18. As a result, the oxidative stress caused by the disequilibrium of ROS production and scavenging can aggravate the inflammatory activity of macrophages via the activation of pro-inflammatory pathways, thus disrupting the homeostasis of cartilage and subchondral bone structure in a vicious cycle. Therefore, scavenging ROS, particularly by modulating mitochondrial metabolic activity, represents a potential strategy for the management of severe joint inflammation.

Recently, nano-therapy has attracted increasing attention for use in the treatment of inflammation, cancer, and metabolic diseases, because of its effective drug encapsulation, prolonged bioavailability and enhanced targeting behavior 19-21. Representing a new class of self-assembled supramolecular nanocarriers, metal-organic supercontainers (MOSCs) based on sulfonylcalix[4]arenes have been designed and synthesized in the past decade 22-25. The multiple binding domains, favorable biocompatibility, stable chemical properties and suitable molecular size for cellular internalization make MOSCs a desirable drug delivery system 24, 26-29. Recently, we reported a magnesium-based MOSC for the enhanced treatment of joint inflammation, which co-loaded target agents and drug molecules in the *exo-* and *endo*-cavities, respectively 30. This design ensured highly efficient synovial accumulation of drug-loaded MOSC and satisfactory anti-inflammatory therapy. However, such designs could not endow MOSCs with the capacity to rapidly overcome the endo/lysosomal barrier and escape into other subcellular compartments for accurate intracellular therapy. Moreover, although various MOSCs have been evaluated in recent years, most of them depend on host-guest effects for sustained drug release and enhanced therapeutic efficacy. The design of a supramolecular drug delivery system with intrinsic ROS-scavenging properties for synergistic anti-inflammatory therapy remains to be determined.

**Figure 1:**
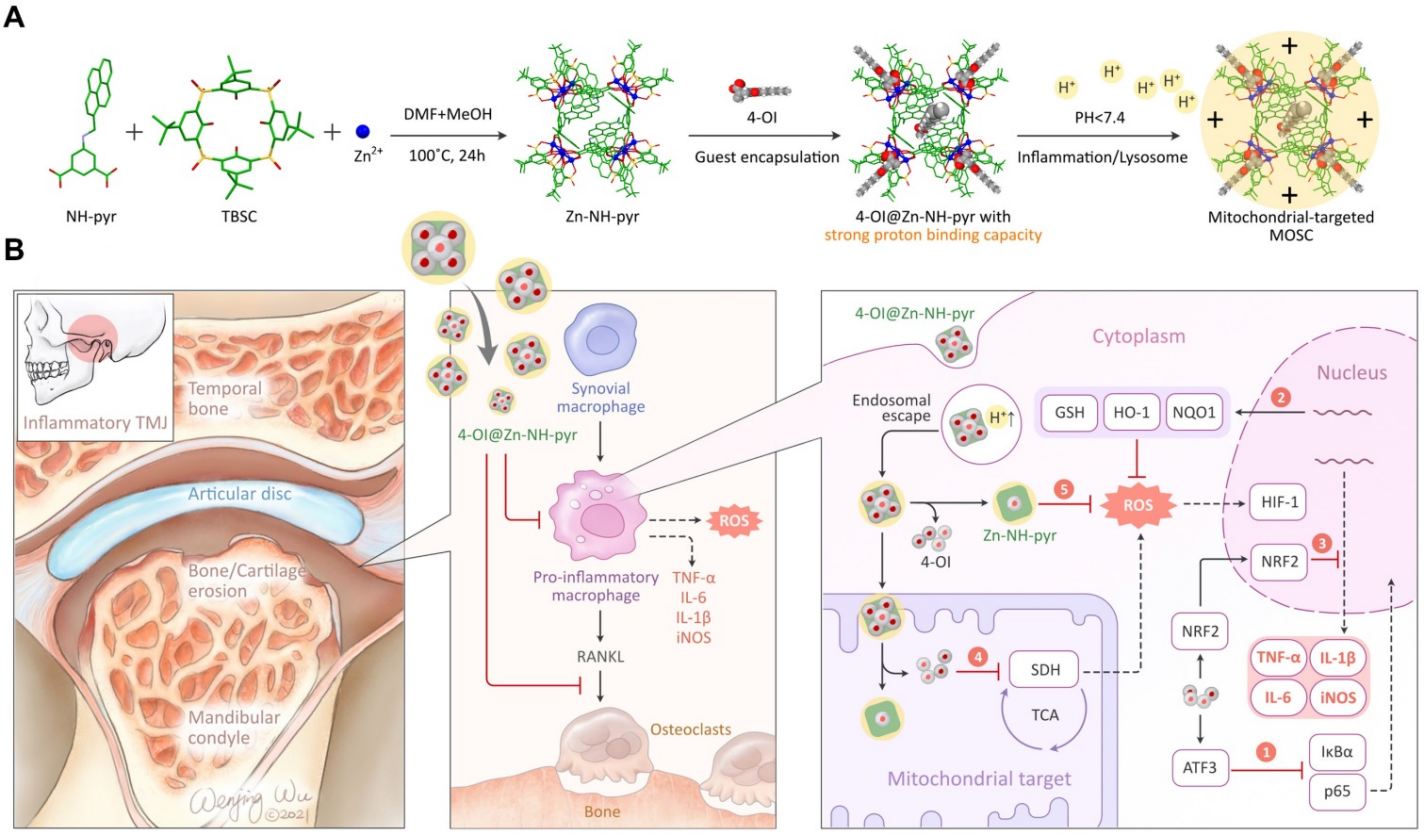
Schematic illustration of the synthesis, encapsulation and application of 4-OI@Zn-NH-pyr for enhanced therapy in severe joint inflammation. (A) Synthesis, encapsulation and protonation of 4-OI@Zn-NH-pyr. (B) Application of 4-OI@Zn-NH-pyr in management of TMJ inflammation by suppressing inflammatory macrophages and OCs. 4-OI@Zn-NH-pyr can alleviate inflammation and ROS production high-efficiently via multiple approaches.

To address these drawbacks, in this study, we selected a zinc-based MOSC (Zn-NH-pyr) with an advantageous proton binding capacity in an acidic environment (inflammation or lysosome) for escaping from the endo/lysosomal barriers and targeting the mitochondria highly efficiently. As electron-rich pools, these MOSCs constructs represent ideal proton receptors, owing to the double proton binding sites in carboxylate linkers including the secondary amine (-NH-) as the principal proton binding site and the pyrenyl units as the additional proton binding site via “cation-π” interaction 31. Such proton-binding capacity makes it unnecessary for traditional triphenylphosphonium (TPP)-based modification by covalent binding or guest encapsulation to escape from the endo/lysosome and target the mitochondria. More importantly, as an electron reservoir with a satisfactory capacity to provide and accommodate electrons, Zn-NH-pyr displays good ROS-scavenging properties, which is immensely beneficial for reinforcing its anti-inflammatory efficacy as a nanocarrier. Furthermore, 4-Octyl itaconate (4-OI), an itaconate derivative, which exerts its anti-inflammatory and anti-oxidative efficacy by inhibiting SDH and activating nuclear factor erythroid 2-related factor 2 (Nrf2), serves as the loaded guest for its enhanced delivery to the mitochondria. In this study, a synergistic therapeutic system for inflammatory macrophages and OCs was developed (Figure 1A). As illustrated in Figure 1B, 4-OI@Zn-NH-pyr was able to alleviate inflammation and ROS levels efficiently via both exogenous and endogenous approaches: (1) the inhibition of NF-κB pathways by 4-OI; (2) the upregulation of antioxidant production via the activation of Nrf2 by 4-OI; (3) the transcriptional repression of pro-inflammatory genes via the activation of Nrf2 by 4-OI; (4) the inhibition of SDH to reduce ROS production by 4-OI, enhanced by the mitochondrial targeting property of Zn-NH-pyr; (5) intrinsic ROS scavenging by Zn-NH-pyr. Both *in vitro* and *in vivo* studies demonstrated that 4-OI@Zn-NH-pyr markedly ameliorated joint inflammation by scavenging excessive ROS, modulating the mitochondrial metabolism of synovial macrophages, and suppressing osteoclast differentiation and function, thus providing a promising curative strategy for severe joint inflammation.

## Materials and Methods

### Reagents and antibodies

The coordination container Zn-NH-pyr was synthesized according to the reported procedure 31. 4-OI was synthesized based on a previously described protocol 32. Bacterial lipopolysaccharide (LPS) of Escherichia coli (tlrl-pb5lps) were purchased from InvivoGen (San Diego, CA, USA). Phorbol-12-myristate-13-acetate (PMA) was purchased from MedChemExpress (NY, USA). *tert*-Butyl hydroperoxide (TBHP) solution was purchased from Macklin (Shanghai, China). Recombinant mouse macrophage colony stimulating factor (M-CSF) and receptor activator of NF-κB ligand (RANKL) were obtained from R&D Systems (Minneapolis, MN, USA). Minimal essential medium alpha (α-MEM) and RPMI-1640 medium was purchased from HyClone (Logan, UT, USA). Fetal bovine serum (FBS) was purchased from Avantor (Radnor, PA, USA). Penicillin was purchased from Gibco BRL (Gaithersburg, MD, USA). The Cell Counting Kit-8 (CCK-8) was obtained from Dojindo Molecular Technology (Rockville, MD, USA). The Prime Script RT reagent Kit and SYBR® Premix Ex Taq™ II were obtained from Takara Biotechnology (Otsu, Shiga, Japan). Primary antibodies against GAPDH (CST #5174), phospho-p65 (CST #3033), p65 (CST #8242), phospho-IκBα (CST #2859), IκBα (CST #4814) as well as secondary antibodies, were purchased from Cell Signaling Technology (CST, Danvers, MA, USA). Primary antibody against iNOS (Thermofisher #PA1-036) was obtained from Thermo Fisher Scientific (Waltham, MA, USA). Primary antibodies against Nrf2 (Affinity #AF0639), HO-1 (Affinity #AF5393) and NQO1 (Affinity #DF6437) were purchased from Affinity Biosciences (Cincinnati, OH, USA). ROS assay kit (S0033), glutathione/glutathione disulfide (GSH/GSSG) assay kit (S0053), Nicotinamide adenine dinucleotide phosphate (NADPH)/NADP+ assay kit (S0179) and 2-(4-Amidinophenyl)-6-indolecarbamidine dihydrochloride (DAPI) (C1002) were obtained from Beyotime Institute of Biotechnology (Haimen, Jiangsu, China).

### Guest encapsulation experiments

The host-guest interaction of Zn-NH-pyr with guest molecules of 4-OI, RB, or TPP was probed using the NMR titration technique. Stock solutions of Zn-NH-pyr and guests (4-OI, RB, or TPP) were prepared in DMSO-*d*_6_ at a concentration of ~2×10^-3^ mol/L and ~0.03 mol/L, respectively. 0.40 mL of the Zn-NH-pyr stock solution was then placed in an NMR tube, to which 2 to 200 μL of the guest solution was added in successive portions. After each addition, the tube was capped and inverted to ensure that the components were fully mixed to reach equilibration. The ^1^H, 2D-DOSY, and 2D-NOESY NMR measurements were collected on a JNM-ECZ400S/L1 spectrometer at 20 °C, with trimethylsilane (TMS) as an internal standard.

To evaluate the overall binding strength, the host-guest chemistry in solutions was probed using the UV-Vis titration technique. Stock solutions of the Zn-NH-pyr were prepared in DMSO at a concentration of ~5×10^-6^ M. 2.00 mL of the stock solution was used to dissolve an accurately known mass of 4-OI, chosen to yield a solution at a concentration 50 - 100 times higher than that of the Zn-NH-pyr. 2.00 mL of the Zn-NH-pyr solution was placed in a 1.0 cm quartz cell, upon which 1 to 500 μL of the 4-OI solution was added gradually. After each addition, the cell was stoppered and inverted. The fluorescent spectra were recorded on a FLS920 fluorescence spectrometer (Edinburgh Instruments). The UV-Vis spectra were collected on a SHIMADZU UV-2600i UV-Vis spectrophotometer at room temperature after 5 min to ensure the complete mixing and equilibration. The titration results were fitted to the non-linear Hill equation 33, 34:



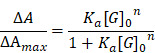



where *ΔA* (= *A*_obs_ - *A*_0_) is the change in absorbance, *ΔA_max_* is the maximum change of absorbance, [G]_0_ is initial guest concentration, *n* is the Hill coefficient, and *K_a_* is the association constant. A plot of 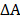 against [*G*]_0_ can be used to estimate *ΔA_max_* and* K_a_*. The titration data were fit to this model using the non-linear regression method by Origin 9 software.

### Extracellular scavenging of ROS

The hydroxyl radicals (HO•) scavenging activity of Zn-NH-pyr was examined via the salicylic acid (SA) method monitored by a UV-Vis spectrophotometer. The HO• was generated by the Fenton reation of FeSO_4_ and H_2_O_2_, while the amount of HO• was monitored by the characteristic absorption peak (510 nm) of 2,3-dihydroxybenzonic acid formed from the oxidation of SA with HO•. Typically, 3 mL of FeSO_4_ (aqueous solution, 2.0 mM), 3 mL of H_2_O_2_ (aqueous solution, 2.0 mM), and 3 mL of SA in ethanol (2.0 mM) were mixed and kept for 15 min, and then 0.5 mL of various concentration of Zn-NH-pyr in DMSO (0.12 - 1.2 mM) was resepctively added into the mixture. Control experments were set up in a similar manner except that the Zn-NH-pyr solutions were replaeced by 4-OI@Zn-NH-pyr or blank DMSO solvents. After incubation for 5 min, the UV-Vis spectra were collected at 25 ℃. The percentage of HO• scavenged was calculated as:

% HO• scavenged = [A_0_ - (A_sample_ - A_blank_)]/A_0_× 100%

where A_0_ is the absorbance at 510 nm of the control solution without Zn-NH-pyr, A_sample_ is the absorbance at 510 nm of the reaction, and A_blank_ is the absorbance at 510 nm of the blank control without H_2_O_2_.

The superoxide radicals (O_2_•^-^) scavenging capacity of Zn-NH-pyr was evaluated by inhibition rate of nitro blue tetrazolium (NBT) photoreduction. Solutions of 0.1 mL of riboflavin (0.2 mM), 0.1 mL of methionine (125 mM), 0.1 mL of NBT (0.75 mM), and 0.1 mL of Zn-NH-pyr in DMSO (7 - 200 μM) were mixed in a PBS solution (pH = 7.4) with a finally solution volume of 2.4 mL. The obtained mixture was illuminated upon UV light for 1.5 min, and then the UV-Vis spectrum was recorded. Control experments were set up in a similar manner except that the Zn-NH-pyr solution was replaeced by 4-OI@Zn-NH-pyr or blank DMSO solvent. The percentage of O_2_•^-^ scavenged was calculated as:

% O_2_•^-^ scavenged = [1 - (A_sample_ - A_0_)/(A_1_ - A_0_)]× 100%

where A_sample_ is the absorbance at 595 nm of the reaction mixture with the addition of Zn-NH-pyr, A_0_ is the absorbance at 595 nm of the control solution before UV illumination, and A_1_ is the absorbance at 595 nm of the control solution after UV illumination.

### Endosomal escape and mitochondrial targeting assay

RAW 264.7 macrophages were seeded in confocal dishes (Cellvis, CA, USA), then incubated with Zn-NH-pyr with or without TPP modification for 1, 12 and 24 h, respectively. Meanwhile, to observe the localization of Zn-NH-pyr in a more visualized approach, RB was encapsulated into Zn-NH-pyr for fluorescent marking, incubated with cells for 3 h. After that, the cells were stained with Lyso-Tracker Green or Mito-Tracker Green (40738ES50 and 40742ES50; Yeasen, China) for another 30 min at 37 ℃. Hoechst 33342 (1:100; C1027; Beyotime Biotechnology, China) was used for nuclear staining in live cells. After several times of rinse with warm PBS to remove excess dyes, the colocalization of Zn-NH-pyr and endo/lysosome or mitochondria were observed in CLSM. The efficacy of endo/lysosomal escape and mitochondrial target was qualitatively analyzed using the Plot Profile instrument by Image J software (National Institutes of Health).

### Intracellular ROS detection

The intracellular ROS was detected using dichloro-dihydro-fluorescein diacetate (DCFH-DA) probe (1:1000; S0033S; Beyotime Biotechnology, China). Briefly, RAW 264.7 macrophages were plated in confocal dishes (Cellvis, CA, USA), cultured with LPS (100 ng/mL) plus various concentrations of Zn-NH-pyr (10, 20 and 40 μg/mL), or various treatment groups (4-OI, Zn-NH-pyr, and 4-OI@Zn-NH-pyr) for 24 h. For THP-1 macrophages, after incubation with PMA for 48 h, the cells were cultured in the presence of various treatments with or without 100 μM TBHP for 24 h. Then the cells were incubated with serum-free culture medium containing 10 μM DCFH-DA for 20 min at 37 ℃. After that, the cells were stained with Hoechst 33342 for nuclear fluorescence, then washed 3 times with warm PBS to remove excess dye. The fluorescent images were captured via CLSM. Image J software was used for semi-quantitative analysis of ROS-positive cells. Meanwhile, flow cytometry was used for quantitative detection of ROS-positive cells in a FACScan flow cytometer (BD, CA, USA), with an excitation wavelength of 488 nm and an emission wavelength of 520 nm. The data were quantified and then presented by FlowJo software (Tree Star, OR, USA).

### Mitochondrial ROS detection

The mitochondrial ROS production was monitored using MitoSox Red probe (40778ES50; Yeasen, China). Briefly, after the 24 h incubation with LPS or TBHP and various treatment groups, cells were cocultured with serum-free culture medium containing 5 μM MitoSox Red probe for 10 min at 37 ℃. Then, the nuclei were stained with Hoechst 33342. After that, the cells were washed 3 times with warm PBS to remove excess dye and observed via CLSM and flow cytometry.

### Intracellular mitochondrial membrane potential assay

The changes of mitochondrial membrane were detected using a JC-1 Mitochondrial membrane potential assay kit (C2006; Beyotime Biotechnology, China). RAW 264.7 macrophages were seeded, then cultured with LPS plus various treatment groups for 24 h. After that, the cells were incubated with JC-1 working solution for 20 min at 37 ℃ and washed with JC-1 staining buffer twice, then observed via CLSM. Image J software was utilized for semi-quantitative analysis of the JC-1 aggregates/monomer fluorescence ratio.

### Analysis of mitochondrial respiration

The Seahorse Xfe96 Extracellular Flux Analyzer (Seahorse Bioscience, MA, USA) was used to measure oxygen consumption rates (OCR) according to the manufacturer's instructions. Briefly, RAW 264.7 macrophages were seeded in Seahorse 96-well plates a density of 1.5 × 10^4^ cells/well. The cells were cultured with LPS plus various treatment groups for 24 h. For THP-1 macrophages, after incubation with PMA for 48 h, the cells were cultured in the presence of various treatments with or without 100 μM TBHP for 24 h. After that, the culture medium was replaced by XF DMEM base medium containing sodium pyruvate (1 mM), Glutamine (2 mM), and Glucose (10 mM). The real time OCR was subsequently detected by sequantial administration of oligomycin (2 μM), FCCP (2 μM) and rotenone&antimycin A (1 μM). The results were normalized by calculating the ratio of OCR to protein concentration.

### Measurement of GSH/GSSG

The intracellular level of GSH/GSSG was measured using GSH and GSSG Assay Kit (S0053; Beyotime Biotechnology, China), according to the manufacturer's instructions. The cells were collected and lysed in a protein-free solution, then frozen and thawed for three repetitively in liquid nitrogen and 37 ℃. After centrifugation, the supernatant of the samples was extracted for measurement of total GSH. The absorbance was detected at 412 nm, and the standard curve was used to quantify the content. The formula was shown as follow: GSH = Total Glutathione - GSSG × 2.

### Measurement of NADPH/NADP+

The intracellular level of NADPH/NADP^+^ was determined using NADP+/NADPH Assay Kit with WST-8 (S0179; Beyotime Biotechnology, China), according to the manufacturer's instructions. The cells were lysed and centrifuged for extraction of the supernatant, which was measured at the absorbance of 450 nm, with the standard curve used for quantification. The formula was shown as follows: [NADP+] = [NADP_total_] - [NADPH].

### Quantitative PCR analysis

To evaluate the expression of inflammation-related genes, RAW 264.7 macrophages were seeded in 6-well plates at a density of 4 × 10^5^ cells/well, then cultured with LPS plus various treatment groups for 24 h. For evaluation of osteoclast genes, bone marrow macrophages (BMMs) were seeded in 6-well plates at a density of 2 × 10^5^ cells/well, then cultured with M-CSF (30 ng/mL) and RANKL (100 ng/mL) for another 4 days, with various treatment groups. Axygen RNA Miniprep Kit (Axygen, Union City, CA, USA) was used for total RNA extraction according to the manufacturer's instructions. After reverse transcription from RNA templates, a real-time PCR (RT-qPCR) assay was performed on an ABI 7500 Sequencing Detection System (Applied Biosystems, Foster City, CA) using the SYBR® Premix Ex Taq™ II. A 10 μL reaction system was established by mixing 5 μL of TB Green, 3 μL of ddH_2_O, 1 μL of cDNA, 0.4 μL of each primer and 0.2 μL ROX Dye2. Cycling conditions were 40 cycles of 95 ºC for 5 s and 60 ºC for 30 s. The specificity of amplification was verified by checking the melting curves. The relative gene expression was calculated using the comparative 2^-ΔΔCT^ method, as described previously. GAPDH was defined as the housekeeping gene. The sequences of the primers are listed in Table S1.

### Western blotting

RAW 264.7 macrophages were seeded in 6-well plates at a density of 4 × 10^5^ cells/well. After adhesion, the cells were incubated with LPS plus various treatment groups for 24 h. Whole cell lysis buffer with protease inhibitor cocktail (P8340; Sigma-Aldrich, Shanghai, China) was used for total protein extraction. The lysate was centrifuged at 12,000 g for 15 min and the protein in the supernatant was collected. Bicinchoninic acid (BCA) assay (P0012; Beyotime Biotechnology, China) was used to measure protein concentrations. Dissolved in loading buffer, the proteins were separated by 10% SDS-PAGE and transferred to 0.22 µm polyvinylidene fluoride (PVDF) membranes. The membranes were blocked in 5% BSA in 1 × TBST (Tris-buffered saline with Tween 20) at room temperature for 1 h, then incubated with the primary antibodies (GAPDH, 1:1000; p-p65, 1:1000; p65, 1:1000; p-IKBα, 1:1000; IKBα, 1:1000; iNOS, 1:1000; Nrf2, 1:1000; HO-1, 1:1000; NQO1, 1:1000) overnight at 4 ºC. Thereafter, the fluorescent secondary antibodies were incubated for 1 h at room temperature and the blots was visualized by Odyssey V3.0 image scanning (Li-COR. Inc., Lincoln, NE, USA).

### Immunofluorescence for macrophages

RAW 264.7 macrophages were stimulated with LPS plus various treatments for 24 h. The cells were fixed, permeabilized and blocked, then incubated with primary antibody against iNOS (1:100; PA1-036; Thermofisher, Waltham, MA, USA). The fluorescent second antibodies were incubated for 1 h. Further, the nuclei were stained with DAPI before imaging. After that, the cells were observed via CLSM, and analyzed by Image J software.

### TRAP staining assay

BMMs were seeded into 96-well plates at a density of 8 × 10^3^ cells/well. After adhesion, the cells were cultured with RANKL (100 ng/mL) and M-CSF (30 ng/mL) in the presence of various treatments. The formation of OCs was commonly observed after 4-day stimulation, which were then fixed with 4% paraformaldehyde (PFA) for 20 min and incubated with the TRAP staining solution at 37 °C for 1 h. TRAP-positive cells with more than three nuclei were counted as OCs. The cells were imaged using an optical microscope (Olympus, Tokyo, Japan) and counted by Image J software.

### Bone resorption assay

BMMs were seeded into 96-well Corning Osteo Assay Surface plates (Corning, NY, USA) at a density of 1 × 10^4^ cells/well. After adhesion, the cells were stimulated with RANKL (100 ng/mL) and M-CSF (30 ng/mL) in the presence of various treatments for 6 days. After that, the mature OCs were eliminated by incubation of 5% sodium hypochlorite for 5 min. The resorption pits were imaged using an optical microscope. The bone resorption area was analyzed by Image J software.

### Immunofluorescence for podosome actin belt

BMMs were cultured in confocal dishes in presence of M-CSF (30 ng/mL) and RANKL (100 ng/mL) with various treatment groups. After the formation of OCs, the cells were fixed, permeabilized, and incubated with TRITC Phalloidin (1:100; CA1610; Solarbio, Beijing, China) for 30 min. Washed with PBS for three times, the nuclei were stained with DAPI for 5 min. After that, the cells were observed via CLSM, and analyzed by Image J software.

### RNA sequencing

RNA sequencing was used to investigate the underlying anti-inflammatory mechanism of Zn-NH-pyr and 4-OI@Zn-NH-pyr. Briefly, RAW 264.7 macrophages were seeded in 6-well plates at a density of 4 × 10^5^ cells/well, then incubated with LPS plus various treatment groups for 24 h. Then, the total RNA was collected using TRIzol reagent, and the total gene expression was examined by the Biomarker Technologies (Beijing, China). GO enrichment analysis and KEGG pathway enrichment analysis were performed using BMKCloud (www.biocloud.net).

### Biodistribution of Zn-NH-pyr

To detect the biodistribution of Zn-NH-pyr, Sulfo-Cyanine7 (Cy7) fluorescent dye (HY-D0824; MedChemexpress, NJ, USA) was encapsulated as the fluorescent label. 50 μL Cy7@Zn-NH-pyr (100 μg/mL) was injected into the left TMJ cavity of Sprague-Dawley (SD) rats (n = 6). 48 h later, 3 rats were sacrificed to evaluate the excretion of Zn-NH-pyr. The rests were monitored by the IVIS Lumina *In Vivo* Imaging System (PerkinElmer Inc, Waltham, MA, USA) immediately and 1, 3, 5 days after the injection, respectively.

To further analyze the biodistribution and metabolism of Zn-NH-pyr in major organs, SD rats (n = 3) were intra-articularly injected with Zn-NH-pyr at a dose of 1 mg/mL (50 μL). One day after injection, rats were sacrificed to harvest the major organs. The tissues were weighed, homogenized and dissolved in aqua regia. Inductively Coupled Plasma Optical Emission Spectrometry (ICP-OES) was used to calculate the percentage of injected dose per gram of tissue (%ID g^-1^).

### Establishment of acute TMJ arthritis

The animal protocol was approved by the Animal Care and Experiment Committee of Ninth People's Hospital Affiliated to Shanghai Jiao Tong University School of Medicine. All experiments were performed according to the guidelines for the Ethical Conduct in the Care and Use of Nonhuman Animals in Research by the American Psychological Association. Acute TMJ Arthritis was established for evaluation of various treatment groups *in vivo*, based upon previous studies.^35^ Briefly, thirty-six 8-week-old male SD rats were randomly divided into five groups: (1) Normal group (9 rats, 3 of which sacrificed on day 0, day 7 and day 14 respectively); (2) Saline group (9 rats, 3 of which sacrificed on day 0, day 7 and day 14 respectively); (3) 4-OI treated group (100 μg/mL; 6 rats, 3 of which sacrificed on day 7 and day 14 respectively); (4) Zn-NH-pyr treated group (equivalent to the concentration loaded with 100 μg/mL 4-OI; 6 rats, 3 of which sacrificed on day 7 and day 14 respectively); (5) 4-OI@Zn-NH-pyr treated group (equivalent to 100 μg/mL 4-OI; 6 rats, 3 of which sacrificed on day 7 and day 14 respectively). The emulsion of complete Freund's adjuvant (CFA, 5 mg/mL; 7023; Chondrex, Redmond, WA, USA) and Chick type II collagen (2 mg/mL; 20012; Chondrex, Redmond, WA, USA) was prepared in terms of the manufacturer's instructions. Thereafter, the emulsion (50 μL) was injected into TMJ cavities of the rats bilaterally. After 3 days, to confirm the establishment of TMJ arthritis, 3 rats from the Normal group or the Saline group were euthanized respectively. Then, various treatment groups were injected intra-articularly, and the injection was performed every 5 days. Rats from each group were euthanized 7 and 14 days after treatment. The joint structures were dissected and photographed, fixed in 4% PFA for 48 h for further analysis.

### Micro-computed tomography

A high-resolution micro-computed tomography (micro-CT, μCT-100, SCANCO Medical AG, Switzerland) was utilized for micro-CT scanning, with the resolution of 10 μm. The X-ray energy was set at 70 kv, 200 μA, with a fixed exposure time of 300 ms.

### Histological and immunohistochemical analysis

After micro-CT scanning, the samples were decalcified in 10% EDTA (pH = 7.4) for 4 weeks. After embedding in paraffin, histological sections were prepared for H&E staining, safranin O-fast green (S&F) staining and TRAP stainging. Synovitis scores, as well as Osteoarthritis Research Society International (OARSI) scores were calculated as previously reports 36, 37. Immunohistochemical (IHC) staining was performed with antibodies against iNOS (1:100; PA1-036; Thermofisher, Waltham, MA, USA) and Nrf2 (1:100; AF0639; Affinity, Cincinnati, OH, USA). The stained slices were photographed under a high-quality microscope (Leica DM4000B). The percentage of positively stained cells in synovium and subchondral bone was quantified by Image J software.

### *In vivo* biosafety evaluation

To evaluate the systemic toxicity of Zn-NH-pyr and 4-OI@Zn-NH-pyr, the organs (heart, liver, spleen, lung and kidney) of rats were also collected for H&E staining 14 days after treatment. Meanwhile, the blood samples were collected for complete blood panel analysis and serum biochemistry analysis.

### Statistical Analysis

All statistical analyses were conducted with a GraphPad Prism 8.0 statistical software package. All data were presented as the mean ± standard deviation (SD). After homogeneity test of variance, differences between two groups were analyzed using unpaired Student's t test (two-tailed). Results for multiple group comparisons were evaluated using one-way analysis of variance (ANOVA) with Tukey's post hoc tests. Significant differences were determined as *P* < 0.05.

## Results and Discussion

### Structure and host-guest chemistry of Zn-NH-pyr

The container Zn-NH-pyr obtained from the solvothermal reaction of Zn(NO_3_)_2_, *p-tert*-Butylsulfonylcalix[4]arene (H_4_TBSC), and 5-((pyren-1-ylmethyl)amino)isophthalic acid (H_2_L) showed the molecular architecture of barrel-shaped boxes 31. It is composed of four TBSC-supported tetranuclear moieties (Zn_4_TBSC) connected to eight pyren-1-ylmethyl-amino functionalized dicarboxylate linkers. Zn-NH-pyr possesses four cup-shaped *exo* cavities (with a diameter of ca. 0.7 nm) from the upper rim of the TBSC, as well as a barrel-shaped *endo* cavity (with a diameter of ca. 1.0 nm) sustained by the tetranuclear moieties and dicarboxylate linkers with two obstructed openings each surrounded by four -NH- connected pyrenyl groups, thus providing unique multiple binding domains for efficient guest encapsulation and drug delivery. Zn-NH-pyr exhibited a strong blue emission band centered at 417 nm accompanied by a shoulder at 466 nm in DMSO solution upon excitation at 405 nm (Figure S1), benefiting the tracking of cellular transportation. The -NH- and pyrene bi-functionalization was increased to as high as 50 equiv. of the proton-binding capacity of Zn-NH-pyr through both amino and pyrenyl units, which potentially enables the container molecule to escape from the endo/lysosome and target the mitochondria 31. The surface potential of Zn-NH-pyr after protonation treatment was examined by zeta potential analysis, which displayed an increase of zeta potential from -22.64 to -9.63 mV, indicating the formation of protonated -NH_2_^+^- groups.

**Figure 2:**
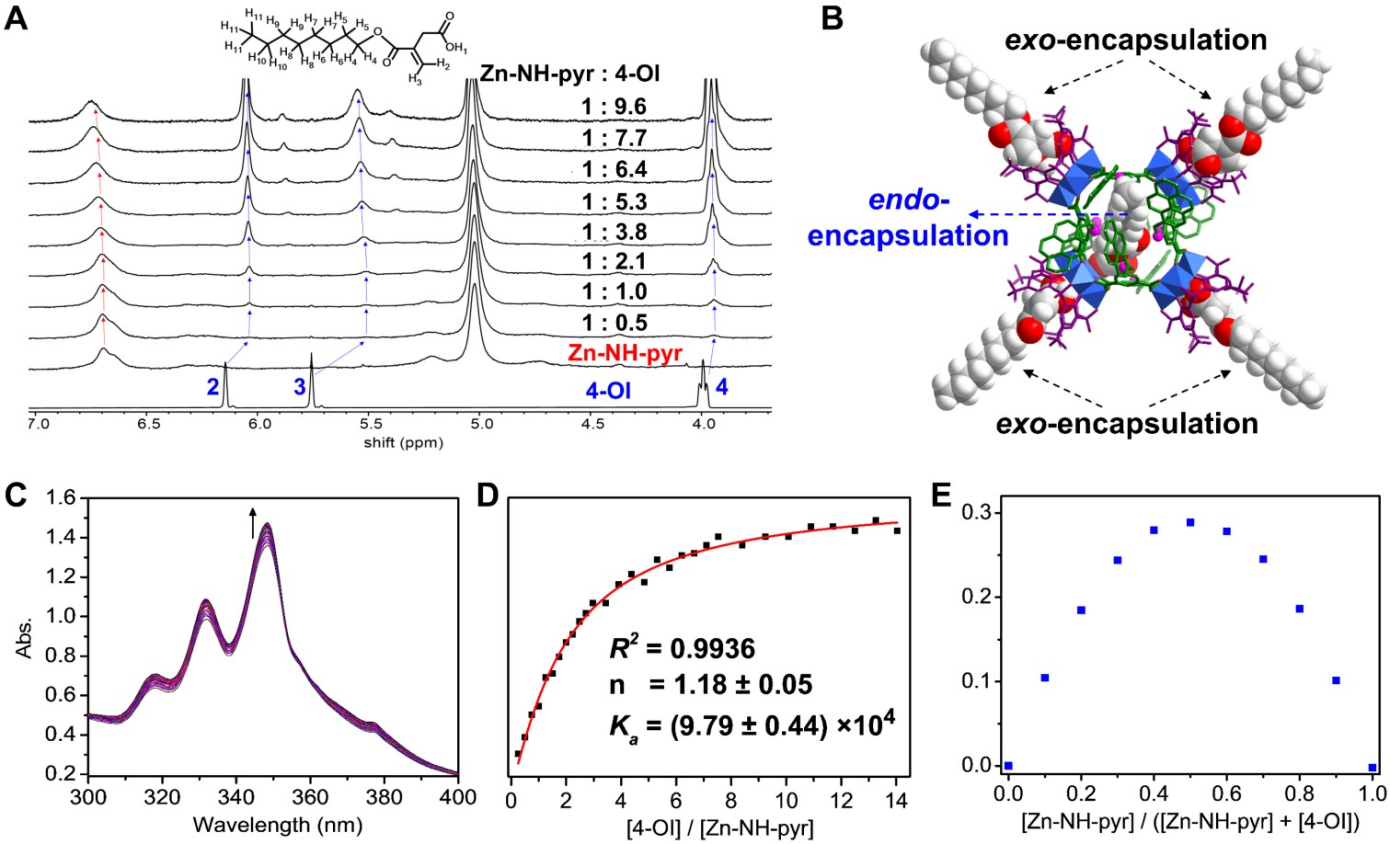
(A) ^1^H NMR spectral changes (400 MHz, DMSO-*d*_6_) of 4-OI titrating into Zn-NH-pyr. (B) Schematic representation of 4-OI encapsulation by *endo* and *exo* cavities of Zn-NH-pyr. (C) UV-Vis spectral changes of Zn-NH-pyr upon the addition of 4-OI. (D) Plot of ∆A vs. 4-OI/Zn-NH-pyr molar ratio based on the UV-Vis spectroscopic titration data with the non-linear fit of the titration data to Hill equation. (E) Job's plot for the determination of the *endo*-encapsulation stoichiometry of Zn-NH-pyr and 4-OI binding, indicating a binding stoichiometric ratio of 1:1.

The guest binding behavior of Zn-NH-pyr was investigated by solution titration experiments using ^1^H NMR and UV-Vis spectroscopic techniques. Upon the addition of 4-OI to a *d_6_*-DMSO solution of Zn-NH-pyr, the chemical shifts of the protons of 4-OI first shifted markedly to a higher field before moving slightly downfield with an increase in the ratio of 4-OI (Figure 2A). The marked upfield shifts of the ethenyl protons (*H_2_* and *H_3_*) indicate that 4-OI is first trapped by Zn-NH-pyr through the *endo* cavity, with the ethenyl unit orientated inside the cavity (vide infra). Further encapsulation of 4-OI through the *exo* cavities of Zn-NH-pyr was unambiguously confirmed by the gradual downfield shifts of the proton signals of 4-OI with increasing stoichiometry ratio of 4-OI. The DOSY NMR (Figure S2) spectral study further demonstrated the formation of only a single host-guest species of 4-OI@Zn-NH-pyr with 1:5 ratio of Zn-NH-pyr (host) to 4-OI (guest). Of the five 4-OI molecules, one was encapsulated in the *endo* cavity and the other four were trapped in the four *exo* cavities of Zn-NH-pyr (Figure 2B).

The binding affinity of 4-OI toward Zn-NH-pyr was further determined by UV-Vis titration experiments. Upon the gradual addition of 4-OI to a DMSO solution of Zn-NH-pyr, the absorption bands of Zn-NH-pyr were gradually enhanced (Figure 2C), clearly suggesting the formation of host-guest complexes between 4-OI and Zn-NH-pyr. The association constant was estimated to be (9.79 ± 0.44) × 10^4^ after fitting the UV-Vis titration data by the Hill equation with a Hill coefficient of n = 1.18 ± 0.05 (Figure 2D), indicating a relatively strong overall binding affinity and a positively cooperative binding behaviour between Zn-NH-pyr and 4-OI. Furthermore, Job's plot suggested a 1:1 *endo*-capsulation stoichiometry of 4-OI by Zn-NH-pyr (Figure 2E).

The stability of Zn-NH-pyr and 4-OI@Zn-NH-pyr in serum was further examined by UV-Vis analysis (Figure S3). As shown in Figure S3C, the absorption intensity of Zn-NH-pyr increased slightly at the beginning, which is probably due to the host-guest interaction between Zn-NH-pyr and serum. In comparison, smaller changes of absorption intensity in 4-OI@Zn-NH-pyr were observed, indicating the stronger binding of 4-OI toward Zn-NH-pyr. Transmission electron microscopy (TEM) observations indicated that Zn-NH-pyr and 4-OI@Zn-NH-pyr had uniformed nanomorphology with a diameter of around 2 nm (Figure S4).

The host-guest interactions between Zn-NH-pyr and TPP (the mitochondria-targeting agent) and Rhodamine B (RB, the fluorescent indicator) were also investigated through ^1^H NMR spectroscopic titration. The aldehyde (*H_1_*) and alkyl protons (*H_2_*, *H_3_* and *H_6_*) of TPP shifted slightly to a higher field immediately after the addition of TPP to Zn-NH-pyr (Figure S5). Meanwhile, the proton signals of RB exhibited a significant upfield shift when titrated with Zn-NH-pyr (Figure S6). The changes in the proton shifts suggested the formation of stable host-guest complexes of TPP@Zn-NH-pyr and RB@Zn-NH-pyr. Moreover, the mutiple-guest encapsulation of Zn-NH-pyr was further confirmed by DOSY NMR spectroscopic studies on the 4-OI/TPP/RB@Zn-NH-pyr system at a molar ratio of Zn-NH-pyr:4-OI:TPP:RB = 1:1:1:3, as shown in Figure S7.

### Extracellular ROS scavenging of Zn-NH-pyr

**Figure 3:**
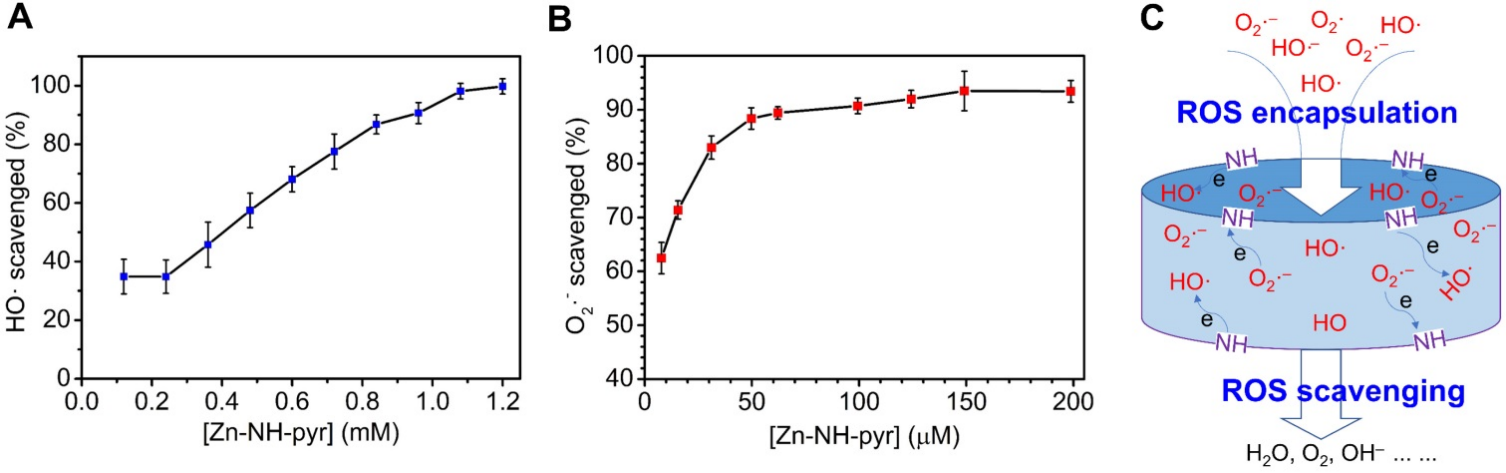
The plots of extracellular ROS scavenging of Zn-NH-pyr. (A) Hydroxyl radicals (HO•) scavenging activity determined at the condition of 3 mL of FeSO_4_ (aqueous solution, 2.0 mM), 3 mL of H_2_O_2_ (aqueous solution, 2.0 mM), and 3 mL of SA in ethanol (2.0 mM) with 0.5 mL of various concentrations of Zn-NH-pyr in DMSO (0.12 - 1.2 mM) (mean ± SD, n = 3 independent samples). (B) Superoxide radicals (O_2_•^-^) scavenging capacity evaluated at the condition of 0.1 mL of riboflavin (0.2 mM), 0.1 mL of methionine (125 mM), and 0.1 mL of NBT (0.75 mM) in a PBS solution (pH = 7.4) with 0.1 mL of various concentrations of Zn-NH-pyr in DMSO (7 - 200 μM) followed by illuminating upon UV light for 1.5 min (mean ± SD, n = 3 independent samples). (C) Proposed mechanism for the ROS scavenging of Zn-NH-pyr.

The well-established catalytic reactivity of MOSCs 38-41 encouraged us to assess the ROS-scavenging activity of Zn-NH-pyr. The extracellular ROS-scavenging ability was evaluated by examining the quenching efficiency of HO• and O_2_•^-^ radical species. As shown in Figure 3A, Zn-NH-pyr exhibited a certain degree of HO• scavenging (~35%), even at a low concentration. Notably, the HO• scavenging capacity increased gradually with the elevated concentrations of Zn-NH-pyr, reaching almost 100% elimination at a Zn-NH-pyr concentration of 1.0 mM. At this point, only 0.08 molar equivalent of Zn-NH-pyr (molar amount of HO• was calculated based on FeSO_4_ in the Fenton reaction) was required to completely remove the radicals, indicating the HO• scavenging capacity of this coordination container. As for O_2_•^-^ scavenging studies, the results (Figure 3B) revealed that Zn-NH-pyr exhibited a prominent O_2_•^-^ scavenging efficiency (~62%) at a concentration of 7.8 μM, and markedly escalated to saturation state (~90%) at Zn-NH-pyr concentration of 100 μM.

To understand the mechanism of ROS scavenging by Zn-NH-pyr, control experiments were performed under similar conditions using the 4-OI@Zn-NH-pyr or dicarboxylate ligand (H_2_L) as the ROS-scavenging reagent. Although the dicarboxylate ligand (L) displays a lower HO• (10%) and O_2_•^-^ (50%) scavenging activity (Figure S8) under the same conditions, it is obvious that the -NH-pyr moiety in the carboxylate ligand is not only a well-known proton accommodating moiety, but also an electron-rich group, thus benefiting radical scavenging by donating the electrons to the highly reactive radicals that prevent the subsequent radical mediated chain reactions of radicals (Figure 3C). Moreover, with the drug molecules encapsulated in the cavities of Zn-NH-pyr, 4-OI@Zn-NH-pyr exhibited slightly lower ROS-scavenging activity as compared to Zn-NH-pyr (Figure S9). Therefore, the much better ROS-scavenging activity of coordination molecular container Zn-NH-pyr is attributable to its inherent and specific characteristics of the structure, including (1) the cavities of Zn-NH-pyr can capture the enriched radicals and then make them in close contact with the -NH- active sites; (2) the incorporation of electron-rich TBSC and pyrene units in Zn-NH-pyr markedly expands the electron-donating and -accommodating capacity of the coordination molecular container; (3) the Brønsted acid μ_4_-H_2_O sites constructed in the tetranuclear cluster units are additional catalytic sources to promote the ROS-scavenging capacity 31.

### Biocompatibility and mitochondria-targeted behaviors of Zn-NH-pyr

**Figure 4:**
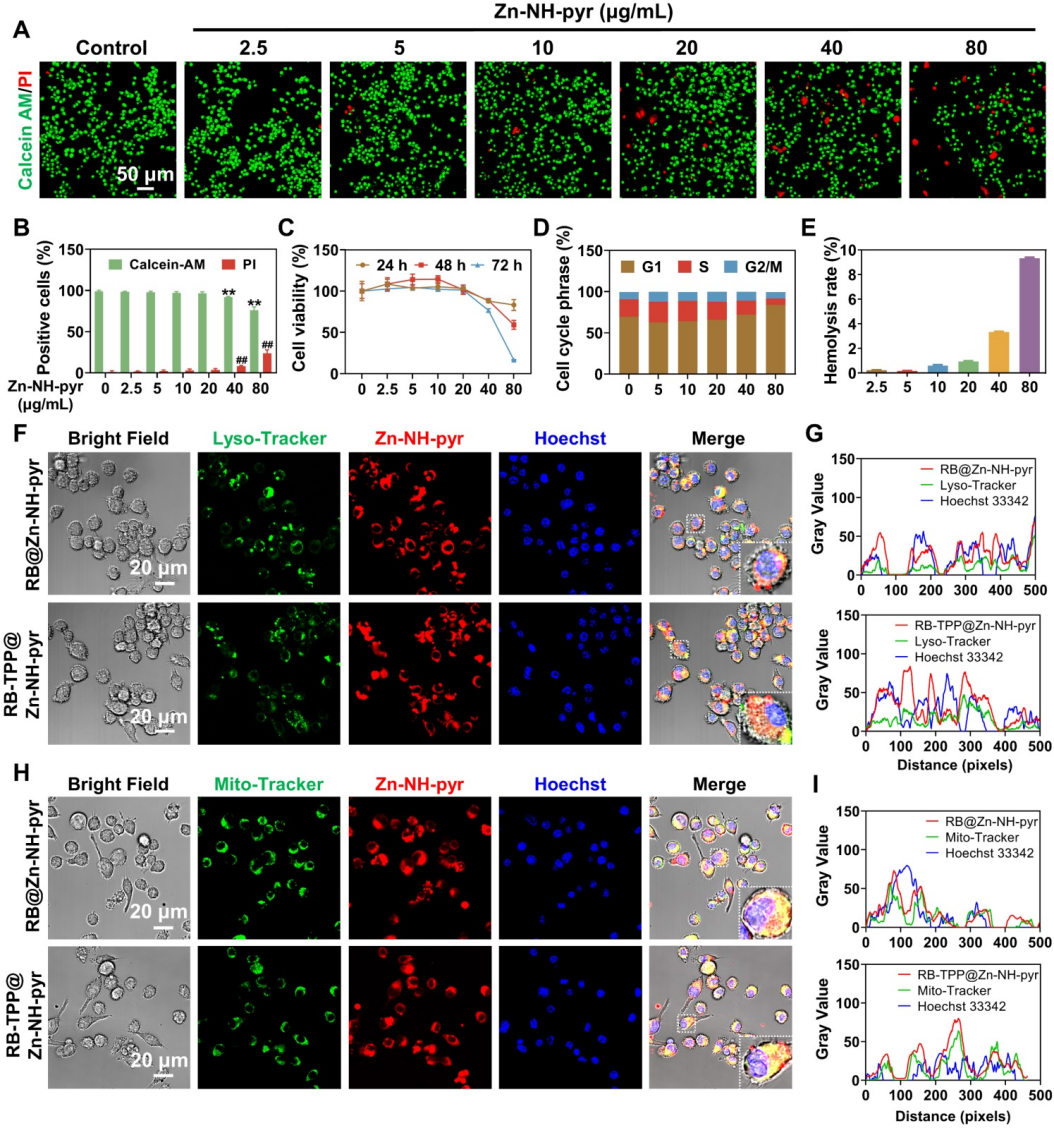
Evaluation of biocompatibility and mitochondria-targeted behaviors of Zn-NH-pyr. (A) Live/dead staining of macrophages treated with various concentrations of Zn-NH-pyr for 24 h (n = 3 independent samples). (B) Quantitative analysis of Calcein-AM and PI positive cells treated with various concentrations of Zn-NH-pyr for 24 h (mean ± SD, unpaired two-tailed Student's t-test, n = 3 independent samples). (C) CCK-8 assay of macrophages treated with various concentrations of Zn-NH-pyr for 24, 48 and 72 h (mean ± SD, n = 3 independent samples). (D) Cell cycle analysis of macrophages treated with various concentrations of Zn-NH-pyr for 24 h. (E) Hemolysis test of Zn-NH-pyr treated with various concentrations of Zn-NH-pyr for 2 h. (F) Confocal images show that similar to RB-TPP@Zn-NH-pyr, most RB@Zn-NH-pyr escaped from the lysosomes 3 h after addition to cells, despite partial colocalization observed (n = 3 independent samples). (G) Qualitative evaluation of colocalization of Zn-NH-pyr and endo/lysosome. (H) Confocal images show that RB@Zn-NH-pyr, as well as RB-TPP@Zn-NH-pyr, accumulated in mitochondria 3 h after addition to cells (n = 3 independent samples). (I) Qualitative evaluation of colocalization of Zn-NH-pyr and mitochondria. ** and ^##^ *P* < 0.01 compared to the 0 μg/mL group.

Although Zn-NH-pyr exhibits proton binding capacity beneficial for mitochondrial targeting as well as ROS-scavenging activity advantageous for anti-inflammation, the biocompatibility of this MOSC should be explored in detail before further *in vitro* and *in vivo* applications. To this end, live/dead staining was performed to visualize the cytotoxicity of Zn-NH-pyr at various concentrations. As shown in Figure 4A-B, Zn-NH-pyr hardly affected the cell viability of RAW 264.7 macrophages when the concentration was 20 μg/mL or below after 24 h of administration. However, increased PI-positive cells and reduced Calcein-AM-positive cells were observed at concentrations of 40 and 80 μg/mL, according to the semi-quantitative analysis of live/dead staining. To further investigate the effect of Zn-NH-pyr on cell viability, a CCK-8 assay was performed after the administration of Zn-NH-pyr for 24, 48 and 72 h. The results showed that Zn-NH-pyr exerted negligible cytotoxicity to RAW 264.7 macrophages at a dose of 20 μg/mL. When the concentration reached 40 μg/mL or higher, cell viability was reduced in a dose- and time-dependent manner (Figure 4C). Similar cytotoxicity results were observed after treatment with 4-OI@Zn-NH-pyr (Figure S10). Flow cytometry demonstrated that Zn-NH-pyr marginally affected the phases of the cell cycle at a dose of 20 μg/mL or below, in line with the cell viability tests (Figure 4D). Furthermore, a hemolysis test was used to assess the hemocompatibility of Zn-NH-pyr, considering the potential effect of nanoscale biomaterials on blood cell homeostasis. As shown in Figure 4E, Zn-NH-pyr negligibly undermined the viability of RBCs at a concentration of 20 μg/mL, with a hemolysis rate lower than 3%. Taken together, these results indicate the satisfactory biocompatibility of Zn-NH-pyr at a dose of 20 μg/mL or below in RAW 264.7 macrophages, which could be utilized without compromising cell activity in the following study.

After determining the safe concentration range, we sought to examine whether Zn-NH-pyr could escape from endo/lysosomal barriers and then target mitochondria with high efficiency. Lyso-tracker and Mito-tracker were used to track the intracellular transportation of Zn-NH-pyr. According to the biocompatibility assays, RAW 264.7 macrophages were treated with Zn-NH-pyr at a concentration of 20 μg/mL. The confocal images demonstrated that Zn-NH-pyr, which exhibited blue fluorescence excited at a wavelength of 405 nm, was endocytosed into cells after 1 h incubation (Figures S11-12). Intriguingly, enhanced fluorescence emission of Zn-NH-pyr was observed after 12 and 24 h incubation, which could be attributed to time-dependent endocytosis and proton-dependent fluorescent enhancement in endo/lysosomes 31. To detect the subcellular location more clearly, RB was loaded into Zn-NH-pyr for the fluorescent labeling. TPP, a cationic moiety widely used for mitochondrial targeting, was encapsulated in Zn-NH-pyr for comparison 42, 43. As shown in Figure 4F, most of the RB@Zn-NH-pyr and RB-TPP@Zn-NH-pyr escaped from the lysosomes 3 h after addition to cells, despite the partial colocalization observed. Qualitative analysis also demonstrated similar endo/lysosomal escape properties of Zn-NH-pyr with or without TPP modification (Figure 4G). Furthermore, both RB@Zn-NH-pyr and RB-TPP@Zn-NH-pyr displayed highly efficient accumulation in the mitochondrial structure (Figure 4H), which was also confirmed by qualitative colocalization analysis (Figure 4I). Consequently, these data suggest that Zn-NH-pyr, a MOSC with proton binding capability, could escape from barriers of the endo/lysosome, then target mitochondria without excess TPP modification.

### *In vitro* scavenging of ROS and mitochondrial metabolic modulation of 4-OI@Zn-NH-pyr

**Figure 5:**
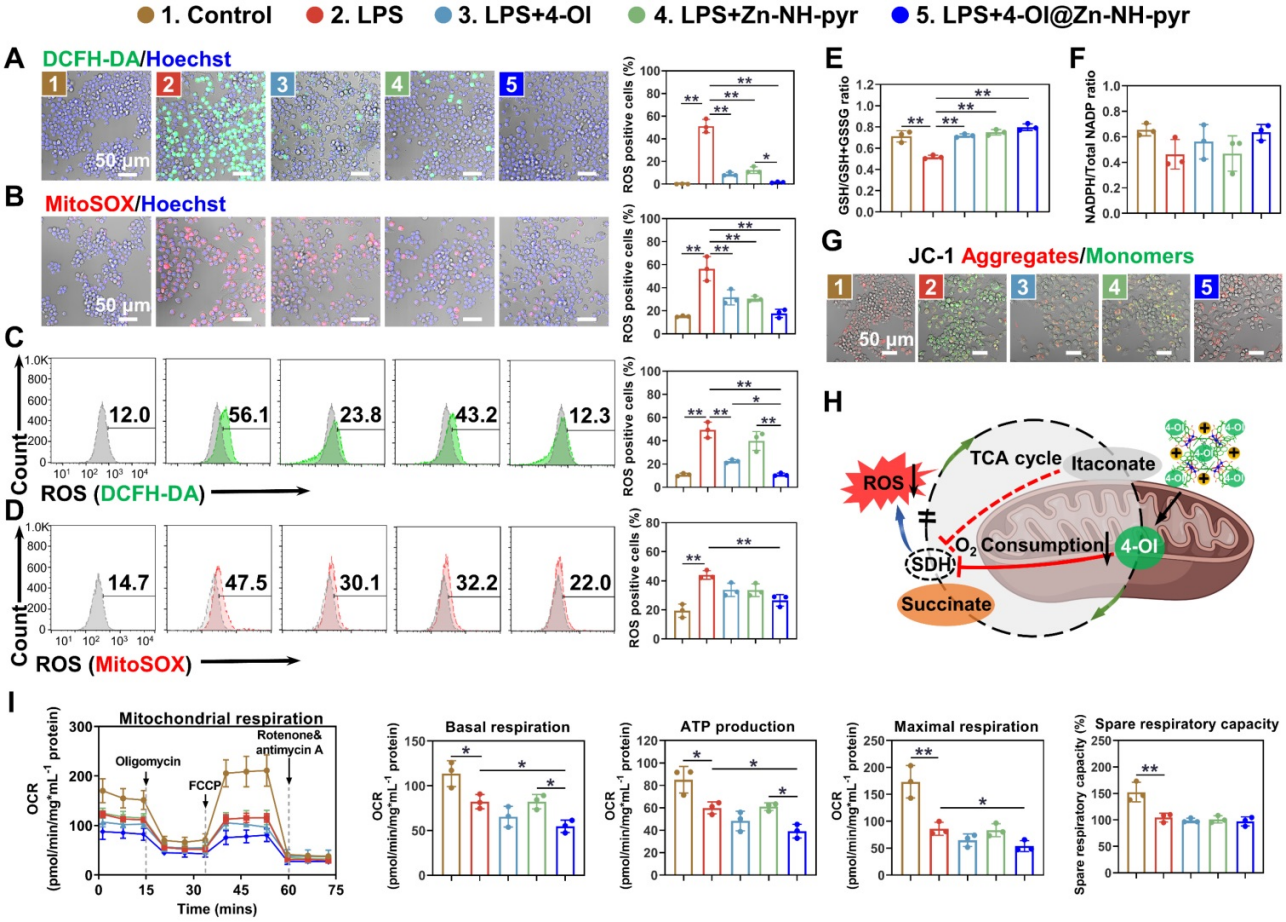
4-OI@Zn-NH-pyr scavenges intracellular ROS and modulates mitochondrial respiration with high efficiency. (A) Confocal images of LPS-activated macrophages from various treatment groups stained with DCFH-DA (mean ± SD, one-way ANOVA with Tukey's multiple comparison test, n = 3 independent samples). (B) Confocal images of LPS-activated macrophages from various treatment groups stained with MitoSOX (mean ± SD, one-way ANOVA with Tukey's multiple comparison test, n = 3 independent samples). (C) Quantification of the percentage of DCFH-DA-positive cells using flow cytometry (mean ± SD, one-way ANOVA with Tukey's multiple comparison test, n = 3 independent samples). (D) Quantification of the percentage of MitoSOX-positive cells using flow cytometry (mean ± SD, one-way ANOVA with Tukey's multiple comparison test, n = 3 independent samples). (E) Intracellular GSH/GSSG levels in LPS-activated macrophages in various treatment groups (mean ± SD, one-way ANOVA with Tukey's multiple comparison test, n = 3 independent samples). (F) Intracellular NADPH/NADP+ level of LPS-activated macrophages with various treatment groups (mean ± SD, one-way ANOVA with Tukey's multiple comparison test, n = 3 independent samples). (G) Confocal images of LPS-activated macrophages from various treatment groups stained with JC-1 mitochondrial membrane potential probe (n = 3 independent samples). (H) Schematic showing the modulation of 4-OI@Zn-NH-pyr on mitochondrial respiration by potent inhibition of SDH. (I) OCR of LPS-activated macrophages in various treatment groups (mean ± SD, one-way ANOVA with Tukey's multiple comparison test, n = 3 independent samples). *P < 0.05; **P < 0.01.

Encouraged by the prominent mitochondrial targeting capacity of Zn-NH-pyr, we next explored the intracellular ROS-scavenging efficacy of Zn-NH-pyr, which was first evaluated in LPS-activated macrophages treated with various concentrations of Zn-NH-pyr (0, 10, 20, and 40 μg/mL). Consistent with the extracellular ROS-scavenging assays, the intracellular ROS level was markedly reduced by Zn-NH-pyr in a concentration-dependent manner (Figures S13-14), indicating the effective ROS-scavenging ability of Zn-NH-pyr at a concentration of ≥ 10 μg/mL. Meanwhile, it was shown that the ligands alone exhibited slight ROS-scavenging capacity, although not as potent as the complete MOSCs (Zn-NH-pyr and Co-NH-pyr) (Figures S15-16). It was reported that 4-OI could downregulate ROS levels by activating Nrf2, thus promoting the expression of various antioxidants, including GSH, HO-1, and NQO1, at concentrations ranging from 5 to 200 μM 44-46. Consequently, the concentration of 4-OI@Zn-NH-pyr was equalized to 5 μM 4-OI in the following study. The DCFH-DA and MitoSOX probes were used to detect intracellular and mitochondrial ROS, respectively. The confocal images demonstrated that the ROS level was significantly depressed with the treatment of 4-OI, Zn-NH-pyr and 4-OI@Zn-NH-pyr in RAW 264.7 macrophages stimulated by LPS (Figure 5A-B), as well as THP-1 macrophages stimulated by TBHP (Figure S17A-B). Flow cytometry was used for the quantitative analysis. As shown in Figure 5C, the percentage of DCFH-DA-positive cells was 49.35 ± 5.51% in LPS-treated group, while reduced to 22.35 ± 1.17%, 39.9 ± 6.66%, and 10.83 ± 1.05% in 4-OI, Zn-NH-pyr, and 4-OI@Zn-NH-pyr treated groups, respectively. Likewise, the percentage of MitoSOX-positive cells was 44.07 ± 2.44% in LPS-challenged group, while this was reduced to 33.8 ± 3.73%, 33.57 ± 3.60%, and 26.60 ± 3.25% in 4-OI, Zn-NH-pyr, and 4-OI@Zn-NH-pyr treated groups (Figure 5D), respectively. In particular, although the single treatment of 4-OI or Zn-NH-pyr shared similar inhibitory effects on intracellular ROS with the treatment of 4-OI@Zn-NH-pyr, the quantitative analysis of flow cytometry demonstrated that 4-OI@Zn-NH-pyr exhibited enhanced ROS-scavenging efficiency in the mitochondria (Figure 5D), which was presumably attributed to the synergy between Zn-NH-pyr and 4-OI. Treatment with 4-OI, Zn-NH-pyr, and 4-OI@Zn-NH-pyr exhibited undetectable effects on intracellular ROS under basal conditions (Figures S18A-B, S19A-B). Furthermore, the intracellular levels of GSH, which serves as an endogenous antioxidant that is excessively consumed in the inflammatory environment, were modestly restored by treatment with 4-OI, Zn-NH-pyr, and 4-OI@Zn-NH-pyr (Figure 5E), indicating reduced oxidative stress. However, no significant improvement was observed in the NADPH levels (Figure 5F). Taken together, these results illustrate the satisfactory ROS-scavenging performance of Zn-NH-pyr in a dose-dependent manner, in addition to the synergistic mitochondrial ROS-clearing performance of 4-OI encapsulated Zn-NH-pyr.

Previous reports have implicated increased mitochondrial ROS production in mitochondrial depolarization, leading to the damaged integrity of the mitochondrial membrane 47, 48. To investigate whether 4-OI@Zn-NH-pyr had a protective effect on mitochondrial membrane integrity, the mitochondrial membrane potential of LPS-activated macrophages was visually detected in various treatment groups. As shown in Figures 5G and S20, when challenged with LPS, the mitochondrial membrane potential of cells was significantly reduced, with increased JC-1 monomers in green fluorescence. However, in the treatment groups, the mitochondrial membrane potential was effectively rescued with reduced JC-1 monomers and increased aggregates, suggesting a protective effect. It was previously reported that itaconate could suppress the production of ROS by mediating mitochondrial respiration in inflammatory macrophages 18, 49. Accordingly, we postulated that 4-OI@Zn-NH-pyr could modulate respiratory activity in mitochondria by the potent inhibition of SDH, thus reducing ROS production with a high efficiency (Figure 5H). To this end, mitochondrial stress test was performed for detection of OCR in inflammatory macrophages (LPS-treated RAW 264.7 macrophages and TBHP-treated THP-1 macrophages) with various treatment groups. Oligomycin, an inhibitor of mitochondrial ATP synthase, was used to measure the ATP production. FCCP, an uncoupler of mitochondrial OXPHOS, was used to measure the maximal and spare respiratory capacity. Rotenone and antimycin A, blockers of electron transport chain (ETC) complexes Ⅰ and Ⅲ, respectively, were used to calculate the non-mitochondrial oxygen consumption. As shown in Figures 5I and S17C, in concert with our postulate, due to the highly efficient mitochondrial targeting efficacy, 4-OI@Zn-NH-pyr exhibited an enhanced inhibitory effect on basal respiration, ATP production and maximal respiration, in comparison with the single 4-OI-treated group, although no significant difference was observed in spare respiratory capacity. Meanwhile, the single Zn-NH-pyr treatment exerted a neglectable influence on OCR. It was also noted that 4-OI@Zn-NH-pyr exhibited inhibition on OCR of both RAW 264.7 macrophages and THP-1 macrophages in the basal condition (Figures S18C and S19C). Taken together, these results demonstrate that 4-OI@Zn-NH-pyr acted as a potent inhibitor of ROS production by targeting the mitochondria of inflammatory macrophages, and then modulating mitochondrial respiration highly efficiently, in addition to its eminent ROS-scavenging capacity.

### Anti-inflammatory and anti-osteoclast activity of 4-OI@Zn-NH-pyr

**Figure 6:**
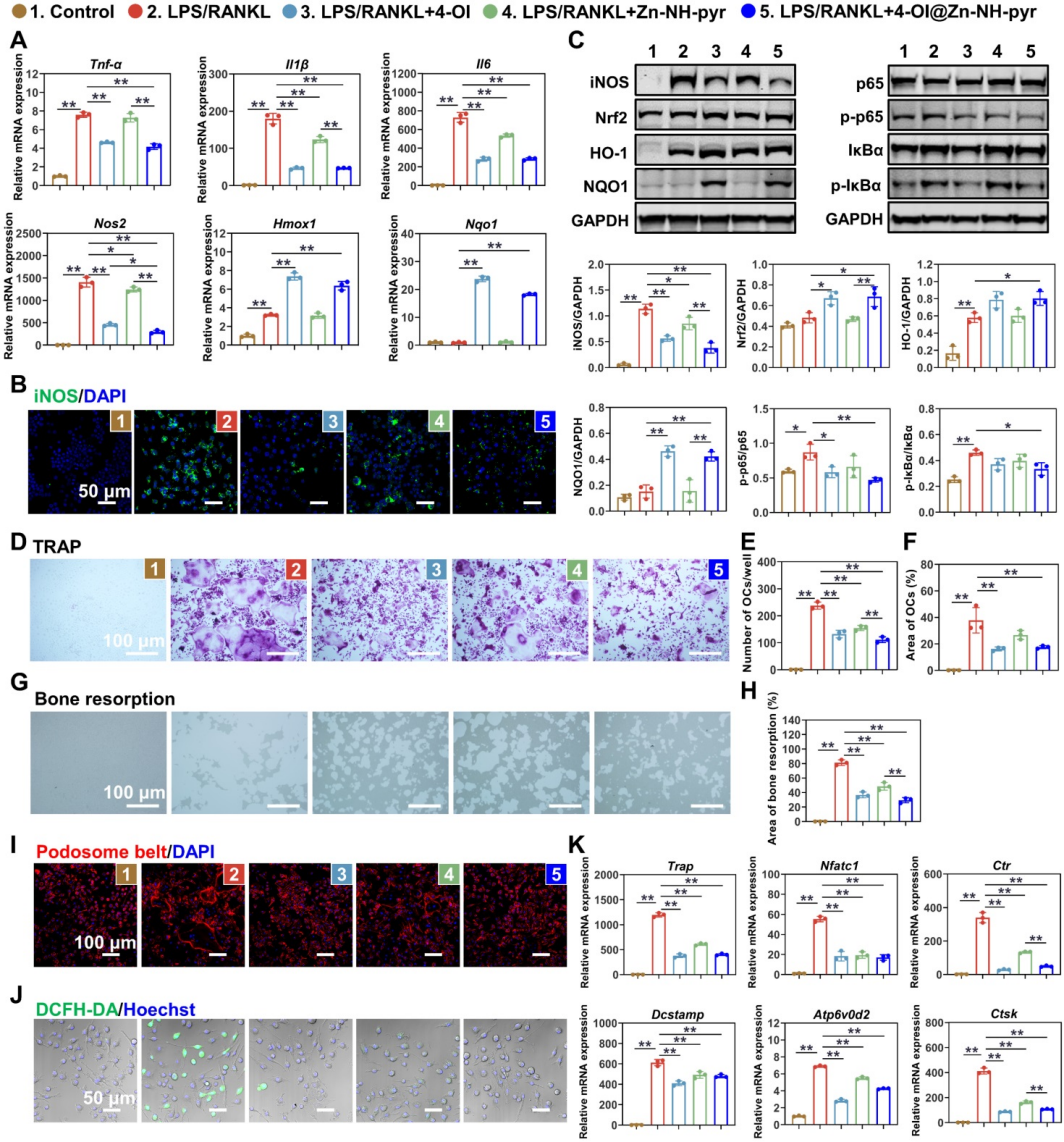
Effect of 4-OI@Zn-NH on inflammatory macrophages and OCs. (A) Expression of pro-inflammatory genes evaluated by RT-qPCR in macrophages (mean ± SD, one-way ANOVA with Tukey's multiple comparison test, n = 3 independent samples). (B) Immunofluorescence of iNOS in LPS-activated macrophages with various treatment groups (n = 3 independent samples). (C) Western blotting showing the expression of Nrf2-related anti-inflammatory pathways and the activation of NF-κB pathways with various treatment groups (mean ± SD, one-way ANOVA with Tukey's multiple comparison test, n = 3 independent samples). (D) TRAP staining of BMMs stimulated by RANKL for 4 days with various treatment groups (n = 3 independent samples). (E) Number of TRAP-positive cells (mean ± SD, one-way ANOVA with Tukey's multiple comparison test, n = 3 independent samples). (F) Area of TRAP-positive cells (mean ± SD, one-way ANOVA with Tukey's multiple comparison test, n = 3 independent samples). (G) Osteo Assay plates showing the bone resorption of OCs with various treatment groups (n = 3 independent samples). (H) Area of bone resorption (mean ± SD, one-way ANOVA with Tukey's multiple comparison test, n = 3 independent samples). (I) Immunofluorescence of podosome belt in RANKL-stimulated BMMs with various treatment groups (n = 3 independent samples). (J) Confocal images of RANKL-stimulated BMMs with various treatment groups stained with DCFH-DA (n = 3 independent samples). (K) Expression of OCs related genes evaluated by RT-qPCR in BMMs stimulated by RANKL for 4 days with various treatment groups (mean ± SD, one-way ANOVA with Tukey's multiple comparison test, n = 3 independent samples). *P < 0.05; **P < 0.01.

After determining the satisfactory ROS-scavenging ability and enhanced modulatory effect of 4-OI@Zn-NH-pyr on mitochondrial respiration, we evaluated the anti-inflammatory efficiency of Zn-NH-pyr and its drug-loaded form. The RT-qPCR results illustrated that the mRNA levels of pro-inflammatory genes, including *Tnf-α*, *Il1β*, *Il6*, and *Nos2*, were upregulated when stimulated with LPS, but significantly reduced after treatment with 4-OI, Zn-NH-pyr, and 4-OI@Zn-NH-pyr (Figure 6A). As expected, the downstream anti-inflammatory genes, *Hmox1* and *Nqo1*, were upregulated after treatment with 4-OI and 4-OI@Zn-NH-pyr. Immunofluorescence also suggested that iNOS expression was effectively downregulated in the various treatment groups (Figures 6B and S21). Notably, it was shown that a single treatment of Zn-NH-pyr also suppressed LPS-induced expression of *Il1β*, *Il6*, and *Nos2* in a dose-dependent manner without affecting *Tnf-α* (Figure S22), indicative of the anti-inflammatory efficacy of Zn-NH-pyr, possibly due to its ROS-scavenging capacity. To evaluate the effects of Zn-NH-pyr and 4-OI@Zn-NH-pyr on macrophages under basal conditions, RT-qPCR was performed in macrophages without LPS stimulation. As shown in Figure S23, the transcription levels of *Il1β*, *Il6* and *Nos2* were markedly downregulated by treatment with 4-OI and 4-OI@Zn-NH-pyr, with an increased expression of anti-oxidative *Hmox1* and *Nqo1*. Single treatment with Zn-NH-pyr resulted in a slight inhibition of *Il1β* and *Il6*, without affecting the mRNA levels of antioxidants, such as *Hmox1* and *Nqo1*. Collectively, these results suggest that 4-OI@Zn-NH-pyr exerts transcriptional repression of pro-inflammatory genes under basal and inflammatory conditions.

To gain further insights into how 4-OI@Zn-NH-pyr inhibited the intracellular inflammatory response, we investigated the molecular mechanism of anti-inflammation by western blotting. It has been reported that the activation of Nrf2 is required for the inhibitory effect of itaconate on inflammation, which increases the production of downstream antioxidants, including GSH, HO-1, and NQO1 44. Herein, we first checked Nrf2 related anti-inflammatory pathways. As shown in Figure 6C, in line with the RT-qPCR and immunofluorescence results, the expression of iNOS was upgraded with LPS stimulation, but greatly reduced in various treatment groups. The expression of Nrf2 and its downstream anti-oxidative products, was upregulated when treated with 4-OI and 4-OI@Zn-NH-pyr. We also evaluated the effects of 4-OI@Zn-NH-pyr on the NF-κB pathway. It was observed that the phosphorylation of p65 and IκBα was activated by the LPS challenge, whereas it was markedly suppressed by treatment with 4-OI and 4-OI@Zn-NH-pyr. Notably, quantitative analysis indicated that the inhibition of the iNOS and NF-κB pathways was more evident in the 4-OI@Zn-NH-pyr-treated group than in the 4-OI-treated group, owing to the synergistic anti-inflammatory effect. Combined with the results above, this demonstrates that 4-OI@Zn-NH-pyr effectively suppressed inflammatory proteins and pathways, with the activation of Nrf2 and its downstream antioxidants.

Inspired by the potent anti-oxidative and anti-inflammatory effects of 4-OI@Zn-NH-pyr, we next investigated the effect of 4-OI@Zn-NH-pyr on RANKL-induced osteoclastogenesis, which requires ROS for differentiation and resorption activity. The cytotoxicity of Zn-NH-pyr toward BMMs was evaluated by the CCK-8 assay, indicating that Zn-NH-pyr exhibited increased cytotoxicity at a dose of 10 μg/mL in BMMs (Figure S24), compared with that in RAW 264.7 macrophages. For the TRAP staining assay, it was observed that a substantial number of TRAP-positive multinuclear OCs were formed after the 4-day stimulation with RANKL (Figure 6D). However, the treatment groups markedly reduced the number and area of OCs (Figure 6E-F). Furthermore, bone resorption activity was evaluated in Osteo Assay plates coated with hydroxyapatite (Figure 6G). The results showed that the area of bone resorption was significantly reduced after treatment with 4-OI, Zn-NH-pyr, and 4-OI@Zn-NH-pyr (Figure 6H). Especially, when treated with 4-OI@Zn-NH-pyr, the bone resorption area was less than 40% of that in the single RANKL stimulated group. Moreover, the cytoskeletal podosome actin belt was examined using immunofluorescence to evaluate the influence of 4-OI@Zn-NH-pyr on the fusion of osteoclast precursor cells. As shown in Figures 6I and S25, treatment with 4-OI, Zn-NH-pyr, and 4-OI@Zn-NH-pyr greatly suppressed podosome actin belt formation, with the majority of mononucleated BMMs detected. Meanwhile, considering the important role of ROS in osteoclast formation and function, we evaluated the ROS-scavenging efficacy of 4-OI@Zn-NH-pyr on osteoclast precursor cells stimulated by RANKL. As expected, the ROS levels in BMMs were notably reduced after treatment with various groups (Figures 6J and S26). To further verify the effect of 4-OI@Zn-NH on OCs at the transcriptional level, RT-qPCR was performed after a 5-day culture of BMMs in an osteoclast-inducing environment. Consistently, the expression of osteoclast genes, including *Trap*, *Nfatc1*, *Ctr*, *Dcstamp*, *Atp6v0d2*, and C*TSK*, was strongly upregulated upon the stimulation with RANKL but considerably reduced after treatment with 4-OI, Zn-NH-pyr, and 4-OI@Zn-NH-pyr (Figure 6K). Collectively, these results indicate that 4-OI, Zn-NH-pyr, and 4-OI@Zn-NH-pyr demonstrated highly efficient inhibitory effects on osteoclast formation, fusion, and function.

### Anti-inflammatory mechanism of Zn-NH-pyr and 4-OI@Zn-NH-pyr evaluated by transcriptome

**Figure 7:**
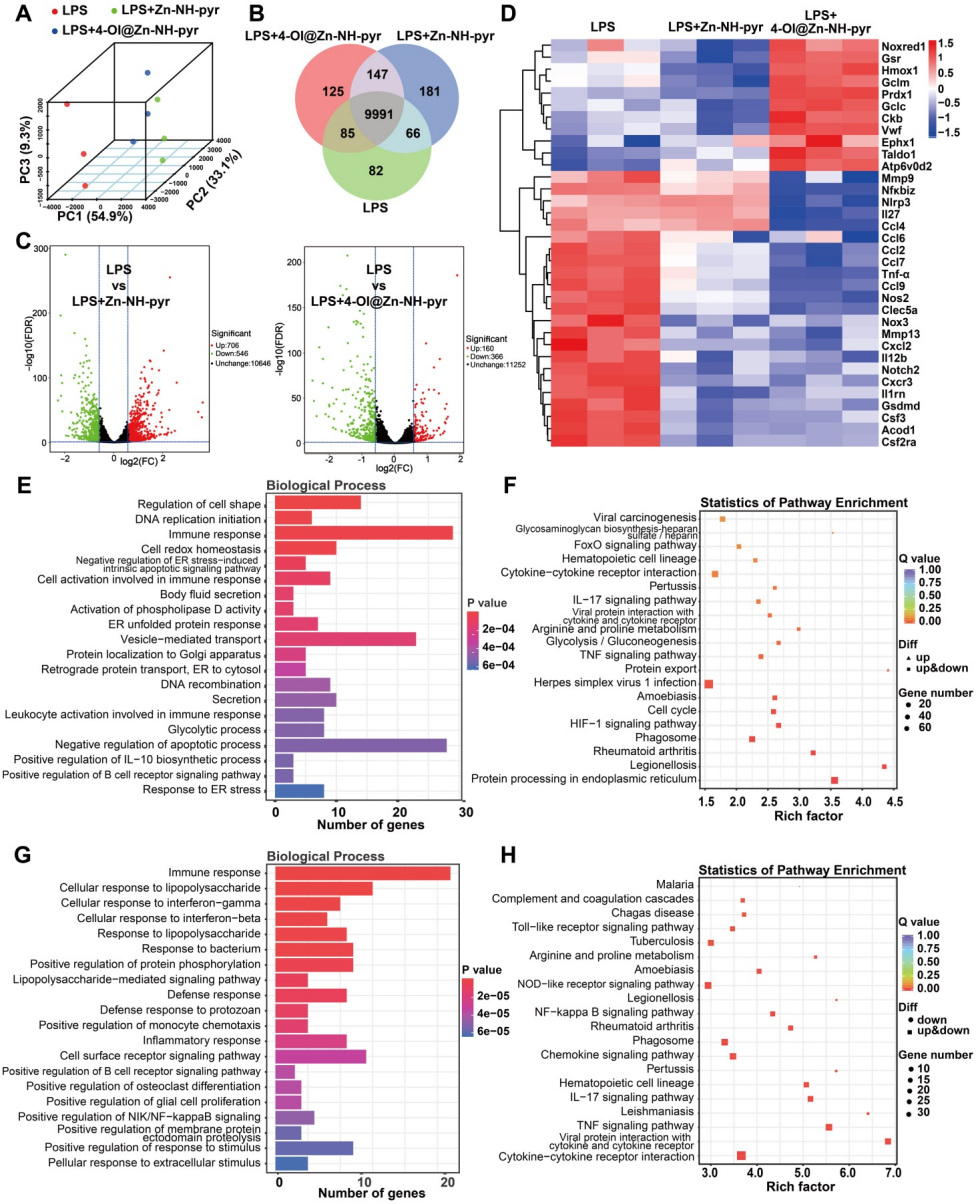
Transcriptomic analysis of the anti-inflammatory mechanism of Zn-NH-pyr and 4-OI@ Zn-NH-pyr. (A) PCA displaying the different transcriptomic profiles among the LPS group, the Zn-NH-pyr treated group and the 4-OI@ Zn-NH-pyr treated group. (B) Venn diagram of gene expression. (C) Volcano plots showing the genes regulated by the treatment of Zn-NH-pyr or 4-OI@ Zn-NH-pyr. (D) Clustered heat map of the representative inflammation-related genes (fold change ≥ 1.5 and P < 0.05). (E) Barplot of biological process of differentially expressed genes between the LPS and the Zn-NH-pyr treated groups. (F) KEGG pathway enrichment analysis on differentially expressed genes between the LPS and the Zn-NH-pyr treated groups. (G) Barplot of biological process of differentially expressed genes between the LPS and the 4-OI@Zn-NH-pyr treated groups. (H) KEGG pathway enrichment analysis on differentially expressed genes between the LPS and the 4-OI@Zn-NH-pyr treated groups.

To gain further insights into the anti-inflammatory mechanism of Zn-NH-pyr and 4-OI@Zn-NH-pyr, and to distinguish between the effects of these two molecules, RNA sequencing was performed in LPS-activated macrophages treated with Zn-NH-pyr or 4-OI@Zn-NH-pyr. Principal components analysis (PCA) showed distinct transcriptomic profiles among the LPS, LPS+Zn-NH-pyr, and LPS+4-OI@Zn-NH-pyr groups (Figure 7A). As illustrated in Figure 7B, the three groups shared 9991 genes, with 82, 181, and 125 genes exclusively expressed in the LPS, LPS+Zn-NH-pyr and the LPS+4-OI@Zn-NH-pyr groups, respectively. Volcano plots demonstrated that 706 genes were significantly upregulated with 546 genes downregulated after treatment with Zn-NH-pyr, while 160 genes were significantly upregulated, with 366 genes downregulated after treatment with 4-OI@Zn-NH-pyr (Figure 7C). As shown in Figure 7D, the heat map displays that treatment with Zn-NH-pyr or 4-OI@Zn-NH-pyr had silimar suppressive effect on various pro-inflammatory gene expression, such as *Il12b*, *Cxcl2*, *Mmp13*, *Nos2* and *Gsdmd*. In addition, 4-OI@Zn-NH-pyr exerted more efficient inhibition than Zn-NH-pyr on a number of pro-inflammatory genes, including *Nlrp3*, *Il27*, *Ccl4*, *Tnf-α*, *Nos2*, and *Nox3*, among others. Notably, treatment with 4-OI@Zn-NH-pyr upregulated the transcriptional expression of a series of Nrf2 downstream antioxidative products, such as *Gsr*, *Hmox1*, *Gclm*, *Prdx1*, *Gclc*, and *Taldo1*, indicating its distinct anti-oxidative effect compared with Zn-NH-pyr. Gene ontology (GO) enrichment analysis was used to investigate the potential effects of Zn-NH-pyr and 4-OI@Zn-NH-pyr on biological processes. As shown in Figure 7E, immune response, cell redox homeostasis, cell activation involved in immune response, positive regulation of IL-10 biosynthetic process, etc., were prominently related to the therapeutic mechanisms of Zn-NH-pyr. Likewise, immune response, cellular response to LPS, cellular response to IFN-γ, cellular response to IL-1β, etc., were highly related to the therapeutic mechanisms of 4-OI@Zn-NH-pyr (Figure 7G). To further investigate the potential signaling pathways affected by Zn-NH-pyr and 4-OI@Zn-NH-pyr treatment, Kyoto Encyclopedia of Genes and Genomes (KEGG) analysis was used to identify the top 20 enriched pathways (with the smallest Q-value). Cytokine-cytokine receptor interaction, IL-17 signaling pathway, glycolysis, TNF signaling pathway, HIF-1 signaling pathway and rheumatoid arthritis were strongly associated with the anti-inflammatory effects of Zn-NH-pyr (Figure 7F). Meanwhile, it was observed that the toll-like receptor signaling pathway, NOD-like receptor signaling pathway, NF-κB signaling pathway, rheumatoid arthritis, chemokine signaling pathway, IL-17 signaling pathway, TNF signaling pathway, and cytokine-cytokine receptor interaction were highly related to the anti-inflammatory effect of 4-OI@Zn-NH-pyr (Figure 7H). Collectively, these data suggest that 4-OI@Zn-NH-pyr may exhibit a wider and stronger anti-inflammatory effect than Zn-NH-pyr by inhibiting pro-inflammatory signaling pathways, such as the NF-κB pathway and NOD-like receptor pathway, and increasing the production of endogenous antioxidants via the activation of Nrf2. In contrast, Zn-NH-pyr may exhibit anti-inflammatory effects by directly scavenging excessive ROS in inflammatory macrophages, regulating subsequent signaling pathways, and inhibiting the secretion of pro-inflammatory molecules.

### *In vivo* biodistribution and therapeutic efficacy of 4-OI@Zn-NH-pyr on TMJ arthritis

**Figure 8:**
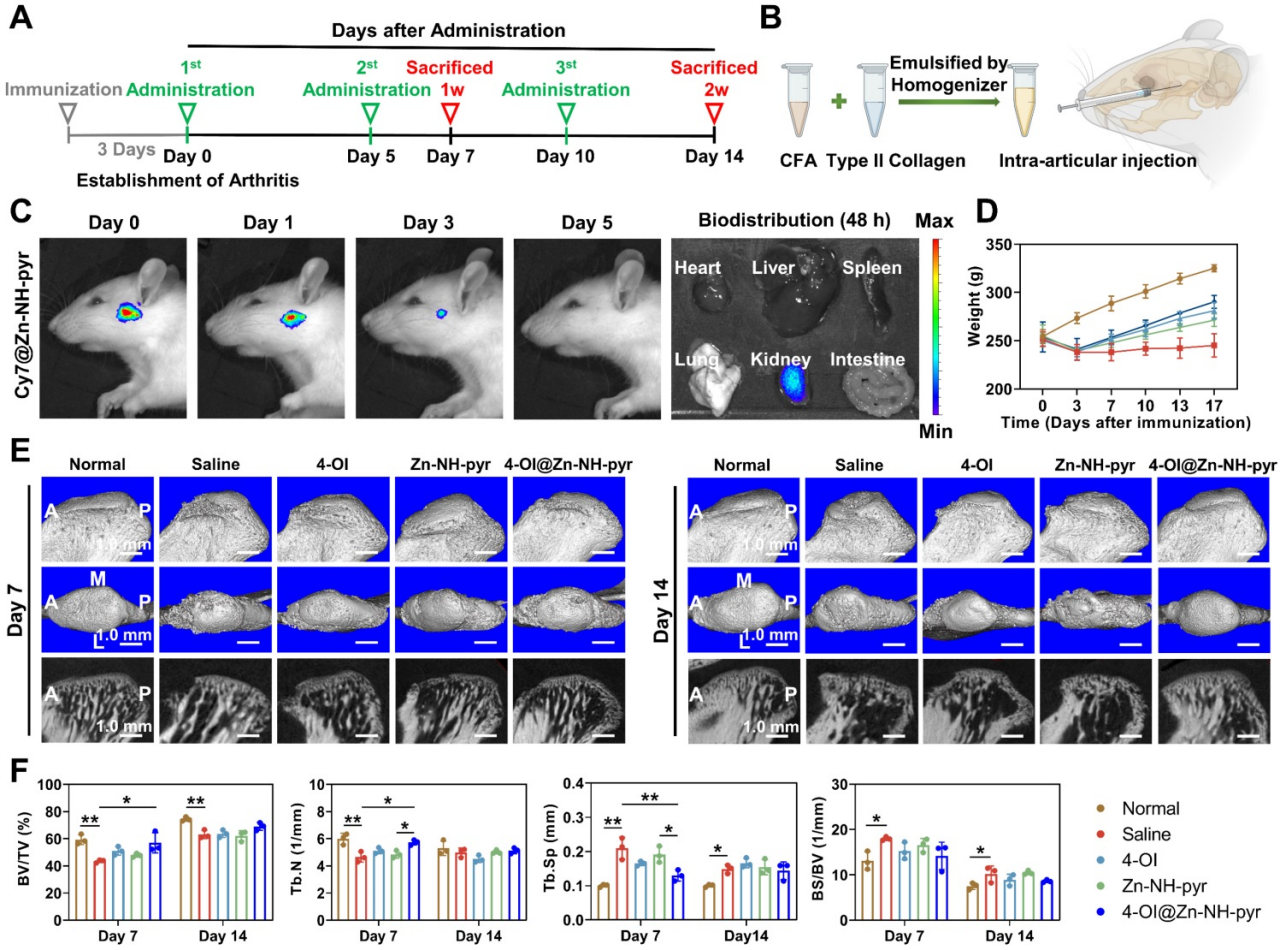
*In vivo* biodistribution and radiological evaluation of 4-OI@Zn-NH-pyr on acute arthritis in TMJ. (A) Schematic diagram of the *in vivo* evaluation. (B) Illustration of preparing emulsion of CFA and type II collagen, and intra-articular injection in rat TMJ. (C) IVIS images of the biodistribution of Cy7@Zn-NH-pyr and its excretion (n = 3 independent animals). (D) Body weight of rats administrated with various treatment groups (mean ± SD, n = 3 independent animals). (E) Representative images of micro-CT scanning of rat TMJ on day 7 and 14 after administration of various treatment groups (n = 3 independent animals). (F) Quantitative analysis of micro-CT scanning of rat TMJ on day 7 and 14 after administration with various treatment groups (mean ± SD, one-way ANOVA with Tukey's multiple comparison test, n = 3 independent animals). *P < 0.05; **P < 0.01.

After determining the satisfactory biocompatibility, mitochondrial targeting, ROS-scavenging and anti-inflammatory effects of 4-OI@Zn-NH-pyr *in vitro*, we sought to explore it *in vivo* therapeutic efficacy by establishing acute arthritis in rat TMJ. A schematic diagram of the *in vivo* evaluation is shown in Figure 8A. An emulsion of CFA and type II collagen was prepared using a homogenizer for the establishment of acute arthritis (Figure 8B). An anterior superior puncture technique of the superior TMJ cavity was performed to ensure accurate intra-articular injection in rats 50. To detect the biodistribution of Zn-NH-pyr, Cy7 was encapsulated in Zn-NH-pyr as a fluorescent label with an outstanding capacity for tissue penetration. After the initial intra-articular injection, the biodistribution of Zn-NH-pyr was tracked using an IVIS system. As shown in Figure 8C, the fluorescence of Cy7@Zn-NH-pyr could be detected in the TMJ immediately, 1 day, or 3 days after the injection, whereas it was undetectable on day 5, indicating that intra-articular administration should be performed every 5 days to maintain the retention of Zn-NH-pyr. Meanwhile, the accumulation of Cy7@Zn-NH-pyr was detected in the kidney 48 h after the initial injection, demonstrating that kidneys may be the excretion pathway of Zn-NH-pyr. Likewise, the results of ICP-OES also demonstrated that kidney exhibited the highest dosage distribution of Zn-NH-pyr, reaching 3.33%ID g^-1^ (Figure S27). This relatively high accumulation in the kidney could be attributed to the ultrasmall diameter (less than 3.0 nm), satisfactory solubility, and discreteness of Zn-NH-pyr, making it possible to be cleared from the blood to renal tubules 51.

After 3 days of injection with the CFA and collagen Ⅱ emulsion, the inflammatory joints were dissected and photographed, with evident swelling and inflammation, indicating the successful establishment of TMJ arthritis (Figure S28). Subsequently, the inflammatory TMJ was administered to various treatment groups every 5 days, according to the observation by IVIS. The rats were sacrificed 7 and 14 days after the first administration to examine their therapeutic effect. In particular, before sacrifice on day 7, the *in vivo* ROS-scavenging capacity was evaluated by intra-articular injection of DCFH-DA. As shown in Figure S29, the fluorescence was intensely enhanced in the Saline group, indicating excessive production of ROS induced by joint inflammation. In contrast, treatment with 4-OI and 4-OI@Zn-NH-pyr significantly reduced the fluorescence level of ROS, which was in line with *in vitro* observations. Notably, it was demonstrated that the treatment of 4-OI@Zn-NH-pyr decreased the fluorescence of ROS more effectively than the single treatment of 4-OI, suggesting its improved *in vivo* ROS-scavenging property. In addition, due to severe TMJ inflammation, it was evident that the rats underwent marked weight loss after immunization with CFA and collagen Ⅱ (Figure 8D). However, various treatments markedly reversed the weight loss by alleviating TMJ symptoms, thus improving food intake.

To further evaluate the therapeutic efficacy, pathomorphological images of inflamed TMJ were obtained, which showed that treatment with 4-OI and 4-OI@Zn-NH-pyr alleviated joint inflammation efficiently, with salved synovial swelling at each time point (Figure S28). Furthermore, micro-CT scanning verified that acute TMJ arthritis was successfully established after the 3-day immunization, with obvious damage to the condylar surface and reduction in BV/TV of the subchondral bone structure (Figure S30). After administration, it was observed that the condylar surface and the subchondral bone structure were effectively protected by the treatment with 4-OI, Zn-NH-pyr and 4-OI@Zn-NH-pyr on days 7 and 14, respectively (Figure 8E). Notably, the 4-OI@Zn-NH-pyr treated group exhibited the most significant improvement in BV/TV and Tb.N on day 7 (after administration), in comparison with those of the other two treatment groups (Figure 8F). On day 14, massive bone remodeling and increased subchondral bone density occurred in the condylar structure, indicating an advanced stage of TMJ inflammation. As expected, minimal condylar remodeling was observed in the 4-OI@Zn-NH-pyr group, with a relatively complete articular surface. Taken together, these data illustrate that 4-OI@Zn-NH-pyr can attenuate severe joint inflammation and protect the subchondral bone structure with high efficiency.

**Figure 9:**
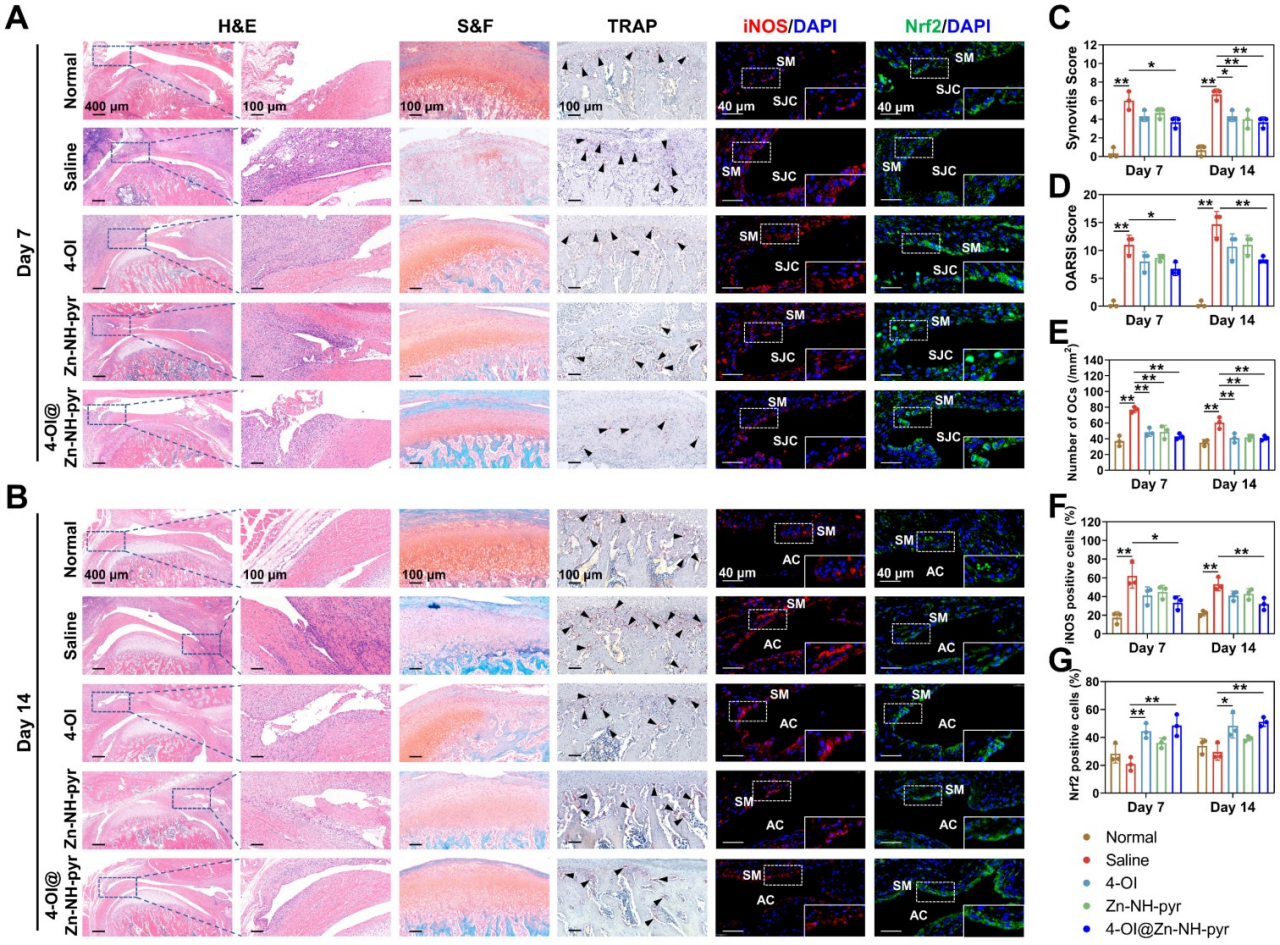
Histological analysis of the therapeutic effect of 4-OI@Zn-NH-pyr on acute arthritis in TMJ. (A) Representative images of H&E, S&F, TRAP, and immunostaining of iNOS and Nrf2 on day 7 after administration of various treatment groups (10 days after immunization) (n = 3 independent animals). (B) Representative images of H&E, S&F, TRAP, and immunostaining of iNOS and Nrf2 on day 14 after administration of various treatment groups (17 days after immunization) (n = 3 independent animals). (C) Quantitative analysis of the synovitis score. (D) Quantitative analysis of the OARSI score. (E) Quantitative analysis of the number of TRAP-positive OCs. (F) Quantitative analysis of the percentage of iNOS-positive cells. (G) Quantitative analysis of the percentage of Nrf2-positive cells. Data are presented as the mean ±SD (n = 3 independent animals). Statistical significance was determined by one-way ANOVA with Tukey's multiple comparison test. Black arrow, TRAP-positive OCs; SM, synovial macrophages; AC, articular cavity. *P < 0.05; **P < 0.01.

For histological evaluation, consistent with the micro-CT scanning, the model of TMJ arthritis was preliminarily established 3 days after immunization, with an inflamed synovial membrane, damaged articular cartilage, and numerous subchondral TRAP-positive OCs (Figure S31). The pathology of joint inflammation was aggravated in another 7 days (10 days after immunization), and was characterized by high-level synovitis and progressive destruction of the condylar structure (Figure 9A). Advanced joint inflammation was observed on day 14 (17 days after immunization), with hyperplastic synovium, a broken cartilaginous surface, and extensive bone erosion of the condyle (Figure 9B). In contrast, treatment with 4-OI, Zn-NH-pyr, and 4-OI@Zn-NH-pyr alleviated OA progression efficiently, with decreased synovitis scores, OARSI scores, and TRAP-positive OCs number in the subchondral bone (Figure 9C-E). Interestingly, the 4-OI@Zn-NH-pyr treated group displayed more significant improvements in synovitis and OARSI scores than the other treatment groups. These results indicate that the condylar cartilage was effectively protected by 4-OI@Zn-NH-pyr owing to the effective inhibition of synovial inflammation. Moreover, histological immunofluorescence was used for the semi-quantitative assessment of inflammatory markers in TMJ inflammation. The results illustrated that the expression of iNOS was markedly elevated in inflammatory synovial tissue, with an accumulation of positive cells in the synovial lining layers. In the treatment groups, the proportion of fluorescence-positive cells was remarkably reduced in the inflammatory synovium, representative of the decreased expression of iNOS (Figure 9F). However, a marked increase in Nrf2-positive cells was detected in the 4-OI and 4-OI@Zn-NH-pyr treated TMJ, indicating the effective activation of Nrf2 by 4-OI treatment (Figure 9G). As expected, taking advantage of the synergistic anti-inflammatory effect of Zn-NH-pyr and 4-OI, the 4-OI@Zn-NH-pyr treated group exhibited the most noticeable inhibition in the expression of iNOS, a typical inflammatory mediator of joint inflammation, thus preventing inflammatory damage to condylar cartilage and attenuating bone resorption activity. Moreover, the biosafety of Zn-NH-pyr and 4-OI@Zn-NH-pyr was examined. H&E staining showed no obvious adverse effects on the histological features of the major organs (Figure S32). In addition, the results of the complete blood panel analysis and serum biochemistry analysis also indicated no obvious toxicity of Zn-NH-pyr and 4-OI@Zn-NH-pyr 14 days after treatment (Figure S33). Collectively, the *in vivo* observations indicate that 4-OI@Zn-NH-pyr markedly attenuated the inflammatory reaction in the TMJ, protecting the condylar structure from severe damage highly efficiently, with favorable biosafety.

## Conclusions

In this study, a zinc-based coordination container with imino-pyrene-functionalized dicarboxylate ligand (NH-pyr) as a proton sponge and electron reservoir with outstanding proton binding capacity, as well as electron-donating and accommodating capabilities in an inflammatory environment, was demonstrated to be a desirable drug carrier for efficient escape from endo/lysosomes and targeting mitochondria, as well as effective ROS scavenging. The ideal proton-binding capacity of Zn-NH-pyr removes the need for traditional TPP-based modifications. As an electron-rich reservoir pool, Zn-NH-pyr exhibits good ROS-scavenging properties owing to its electron-donating and accommodating capabilities. One of the exogenous itaconates, 4-OI, was encapsulated effectively into a zinc-based coordination container for the construction of a novel synergistic therapeutic system for inflammatory macrophages and OCs. Both *in vitro* and *in vivo* studies demonstrated that 4-OI@Zn-NH-pyr can markedly ameliorate joint inflammation by eliminating the overproduced ROS and modulating mitochondrial metabolism in synovial macrophages, in addition to suppressing osteoclast differentiation and function. By evaluating a zinc-organic supercontainer (Zn-NH-pyr) with ideal proton-accepting and electron-accommodating capacity as a drug-loaded matrix for the first time, this study paves the way for the management of severe joint inflammation by designing supramolecular drug-delivery systems with subcellular targeting and ROS-scavenging activity.

## Supplementary Material

Supplementary methods, figures and table.

## Abbreviations

OA: osteoarthritis
RA: rheumatoid arthritis
TMJ: temporomandibular joint
NSAIDs: non-steroidal anti-inflammatory drugs
DMARDs: disease-modifying anti-rheumatic drugs
OCs: osteoclasts
TNF-α: tumor necrosis factor-α
IL: interleukin
iNOS: inducible nitric oxide synthase
ROS: reactive oxygen species
OXPHOS: oxidative phosphorylation
TCA: tricarboxylic acid
SDH: succinate dehydrogenase
MOSCs: metal-organic supercontainers
TPP: triphenylphosphonium
4-OI: 4-Octyl itaconate
Nrf2: nuclear factor erythroid 2-related factor 2
LPS: lipopolysaccharide
M-CSF: macrophage colony stimulating factor
RANKL: receptor activator of NF-κB ligand
PMA: Phorbol-12-myristate-13-acetate
TBHP: *tert*-Butyl hydroperoxide
α-MEM: minimal essential medium alpha
FBS: fetal bovine serum
CCK-8: Cell Counting Kit-8
GSH/GSSG: glutathione/glutathione disulfide
NADPH/NADP^+^: Nicotinamide adenine dinucleotide phosphate
DAPI: 2-(4-Amidinophenyl)-6-indolecarbamidine dihydrochloride
HO•: hydroxyl radicals
O2•^-^: superoxide radicals
NBT: nitro blue tetrazolium
OCR: oxygen consumption rates
PVDF: polyvinylidene fluoride
PFA: paraformaldehyde
CFA: complete Freund's adjuvant
S&F: safranin O fast green
OARSI: Osteoarthritis Research Society International
ANOVA: one-way analysis of variance
H4TBSC: *p-tert*-Butylsulfonylcalix[4]arene
RB: Rhodamine B
PCA: principal components analysis
GO: Gene ontology
KEGG: Kyoto Encyclopedia of Genes and Genomes
GSEA: gene set enrichment analysis
