# Supplementary material for: Mitochondria-targeted supramolecular coordination container encapsulated with exogenous itaconate for synergistic therapy of joint inflammation
Source: Theranostics. 2022 Apr 4;12(7):3251–72. doi: 10.7150/thno.70623 (PMC9065189; doi:10.7150/thno.70623)
Supplement: Supplementary file 1 — Supplementary methods, figures and table. [file thnov12p3251s1.pdf]

Supporting Information

Mitochondria-targeted supramolecular coordination container encapsulated with exogenous itaconate for synergistic therapy of joint inflammation

Xuzhuo Chen[a,1], Chang Li[b,1], Xiankun Cao[c,1], Xinlin Jia[c], Xinwei Chen[a], Zhenqiang Wang[d], Weifeng Xu[a,*], Fengrong Dai[b,*], Shanyong Zhang[a,*]

[a]*Department of Oral Surgery, Shanghai Key Laboratory of Stomatology & Shanghai Research Institute of Stomatology, National Clinical Research Center for Oral Diseases, Shanghai Ninth People's Hospital, College of Stomatology, Shanghai Jiao Tong University School of Medicine, Shanghai, 200011, China*

[b]*State Key Laboratory of Structural Chemistry, Fujian Institute of Research on the Structure of Matter, Chinese Academy of Sciences, Fuzhou, Fujian, 350002, China*

[c]*Department of Orthopedics, Shanghai Key Laboratory of Orthopedic Implant, Shanghai Ninth People's Hospital, Shanghai Jiao Tong University School of Medicine, Shanghai, 200011, China*

[d]*Department of Chemistry & Center for Fluorinated Functional Materials, University of South Dakota, Vermillion, South Dakota, 57069-2390, United States*

[*]Corresponding authors.

E-mail addresses: xwf19891215@163.com (WF. Xu), dfr@fjirsm.ac.cn (FR. Dai), zhangshanyong@126.com (SY. Zhang).

[1]These authors contributed equally.

The supporting information includes:

- Supplementary Methods
- Supplementary Figures
- Supplementary Tables

Supplementary Methods

General methods

^1H NMR spectra were recorded on a Bruker 400 MHz instrument with trimethylsilane (TMS) as internal standard. 2D DOSY NMR and NOESY experiments were performed on JNM-ECZ400S/L1 spectrometer at 20 °C. UV–Vis absorption spectra were measured on a Perkin-Elmer Lambda 35 UV–Vis spectrophotometer. The excitation and emission spectra were collected using an Edinburgh FLS-920 fluorescence spectrometer. Zeta potentials were measured by a laser particle size and zeta potential analyzer (Brookhaven, BI-200SM). The morphology of the material was observed by a field emission transmission electron microscope (FETEM, FEI Tecnai F20).

Cell cultures

RAW 264.7 macrophages, purchased from the Stem Cell Bank of Chinese Academy of Sciences (Shanghai, China), were cultured in complete α-MEM with 10% FBS, 100U/mL

penicillin and streptomycin at 37 °C with 5% CO_2 in a humid environment. When the confluence were approximately 80 – 90%, the cells were ready to be seeded or passaged. To avoid additional stimuli to the macrophage cell line, scrapers were used to remove the attached cells instead of trpsin, in the process of cell dissociation.

The mice primary bone marrow macrophages (BMMs) were obtained and cultured as previous protocols [1]. Briefly, the cells were isolated from the femurs and tibiae of 4-week-old C57/BL6 male mice, then suspended in complete α-MEM with 30 ng/mL M-CSF at 37 °C with 5% CO_2 in a humid environment. The culture medium was refreshed every 2 days with the supplement of 30 ng/mL M-CSF. When the confluence were approximately 80-90%, the primary BMMs were ready to be seeded, dissociated by 0.25% Trpsin-EDTA (Gibco BRL, Gaithersburg, MD, USA).

Human THP-1 monocytic cell line was purchased from the Stem Cell Bank of Chinese Academy of Sciences (Shanghai, China). The cells were cultured in RPMI-1640 medium containing 10% FBS at 37 °C in 5% CO_2 atmosphere. For differentiation into macrophages, the THP-1 cells were incubated with 50 nM PMA for 48 h.

Cytotoxicity assay

For live/dead staining, RAW 264.7 macrophages were treated with various concentrations of Zn-NH-pyr for 24 h. After tbhp, the cells were incubated with PI solution and Calcein-AM for 15 min. Then the live/dead cells were observed via confocal laser scanning microscopy (CLSM, Leica TCS-SP5, DM6000-CFS). The results were quantified by calculating the percent positive cells of Calcein-AM and PI, respectively.

For quantitative CCK-8 assay, RAW 264.7 macrophages and BMMs were seeded into 96-well plates at a density of 8 × 10^3 cells/well, respectively. Treated with various concentrations of Zn-NH-pyr, the cells were incubated for 24, 48, and 72 h. 10 μL of CCK-8 solution was added to each well in every time point. After 2 h incubation, the absorbance was measured at the wavelength of 450 nm, with 630 nm as the reference wavelength in a microplate reader. The quantitative results were demonstrated as cell viability relative to the control group, which was set at 100%.

Hemolysis test

Fresh blood was obtained from 4-week-old C57/BL6 male mice in anticoagulated tubes, centrifuged and washed until the supernatant was clear. After dilution with PBS, the red blood cells (RBCs) suspension was added into tubes in the same volume, with various concentrations of Zn-NH-pyr. Double distilled water and PBS were used as positive and negative controls, respectively. After 2 h incubation at 37 °C and centrifugation at 3,000 rpm for 5 min, the absorbance of the supernatant of each tube was measured at 540 nm by a microplate reader. The calculation of hemolytic rate was shown as follows: Hemolytic rate (%) = $(OD_{sample}-OD_{negative})/(OD_{positive}-OD_{negative}) \times 100\%$.

Cell cycle analysis

For evaluation of cell cycle distribution, RAW 264.7 macrophages were incubated with various concentrations of Zn-NH-pyr for 24 h. After treatment, cells were collected and fixed in cold 70% ethanol at 4 °C for 30 min. Then the cells were centrifuged at 1000 rpm for 5 min, and incubated with 50 μg/mL PI (Beyotime, Shanghai, China) for 30 min. Subsequently, the

cell cycle was analyzed by a FACScan flow cytometer (BD, CA, USA) for at least 20,000 cells per sample, then processed by FlowJo software.

Intracellular superoxide detection

The intracellular superoxide was detected using Dihydroethidium (DHE) (HY-D0079; MedChemExpress, China). Briefly, RAW 264.7 macrophages were cocultured with 5 μM DHE for 30 min at 37 °C after the 24 h incubation with LPS and various treatment groups. Then, the nuclei were stained with Hoechst 33342. After that, the cells were washed 3 times with warm PBS to remove excess dye and observed via CLSM.

Measurement of ROS by microplate

RAW 264.7 macrophages were seeded in 96-well plate (1×10^4 cells/well), then incubated with various treatment groups for 24 h. For THP-1 macrophages, after incubation with PMA for 48 h, the cells were cultured in the presence of various treatments with or without 100 μM TBHP for 24 h. After that, the cells were stained with serum-free culture medium containing 10 μM DCFH-DA for 20 min at 37 °C, then washed 3 times with warm PBS. The cellular fluorescence intensity was detected in a microplate reader (excitation wavelength: 488 nm, emission wavelength: 525 nm). DCFH-DA fluorescence intensity was presented as the percentage relative to the value in Control group.

Supplementary Figures

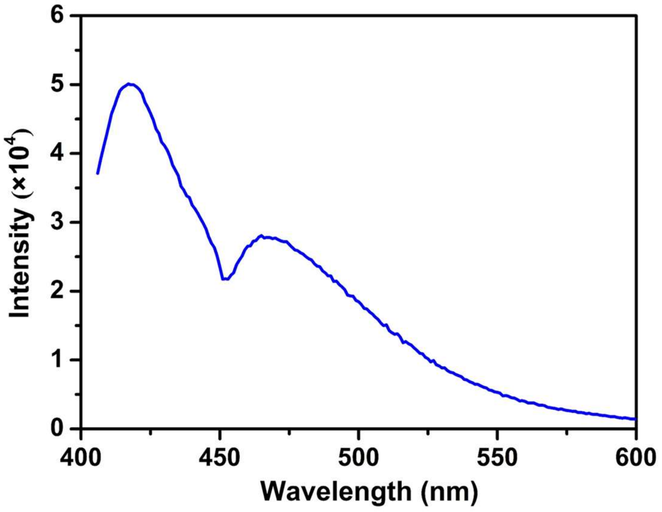

Figure S1. The emission spectrum of Zn-NH-pyr in DMSO solution upon excitation at 405 nm.

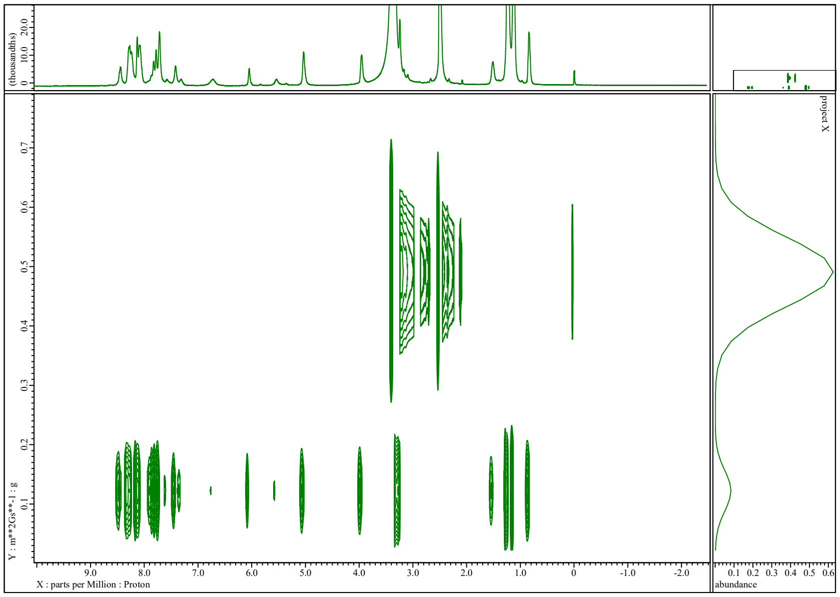

Figure S2. The DOSY NMR (400 MHz, DMSO-D_6, 25 °C) spectrum of 4-OI@Zn-NH-pyr at a ratio of Zn-NH-pyr:4-OI = 1:5.

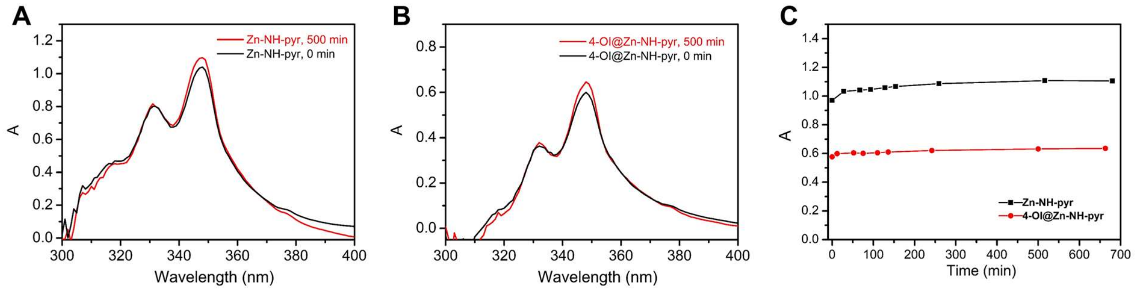

Figure S3. UV–Vis absorption spectra of (A) Zn-NH-pyr and (B) 4-OI@Zn-NH-pyr in serum collected at different durations. (C) Plots of UV–Vis absorption intensity vs. time for Zn-NH-pyr and 4-OI@Zn-NH-pyr in serum.

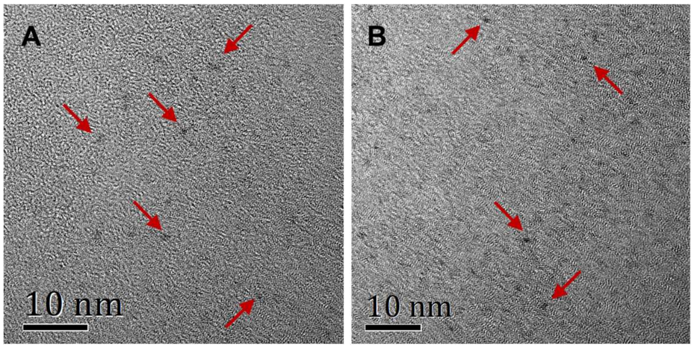

Figure S4. TEM images obtained from aqueous solution of (A) Zn-NH-pyr and (B) 4-OI@Zn-NH-pyr.

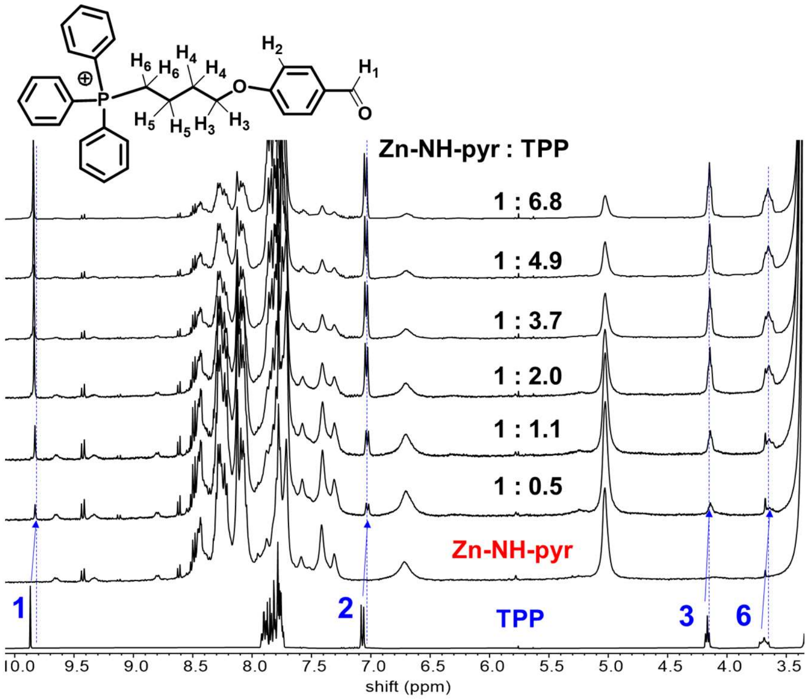

Figure S5. The ^{1}H NMR spectral changes (400 MHz, DMSO-d_6) of TPP during the titration experiment with Zn-NH-pyr.

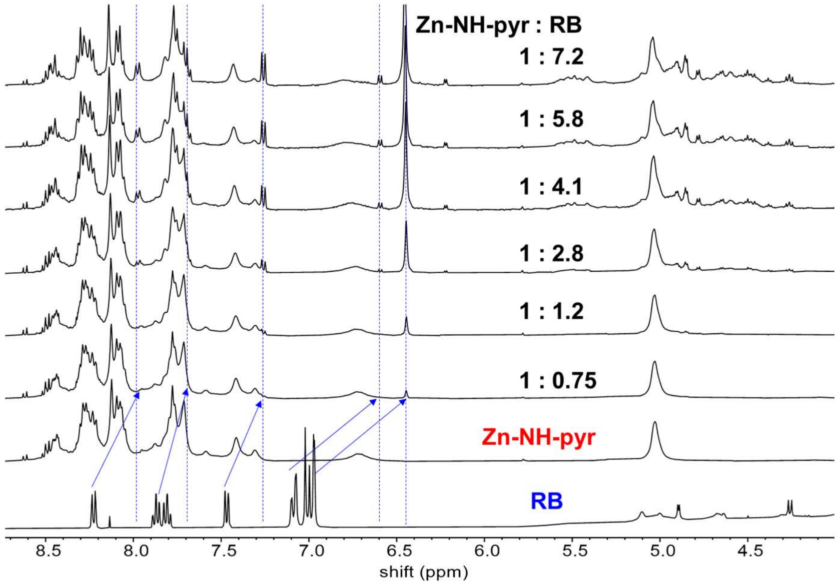

Figure S6. The ^{1}H NMR spectral changes (400 MHz, DMSO-d_6) of RB during the titration experiment with Zn-NH-pyr.

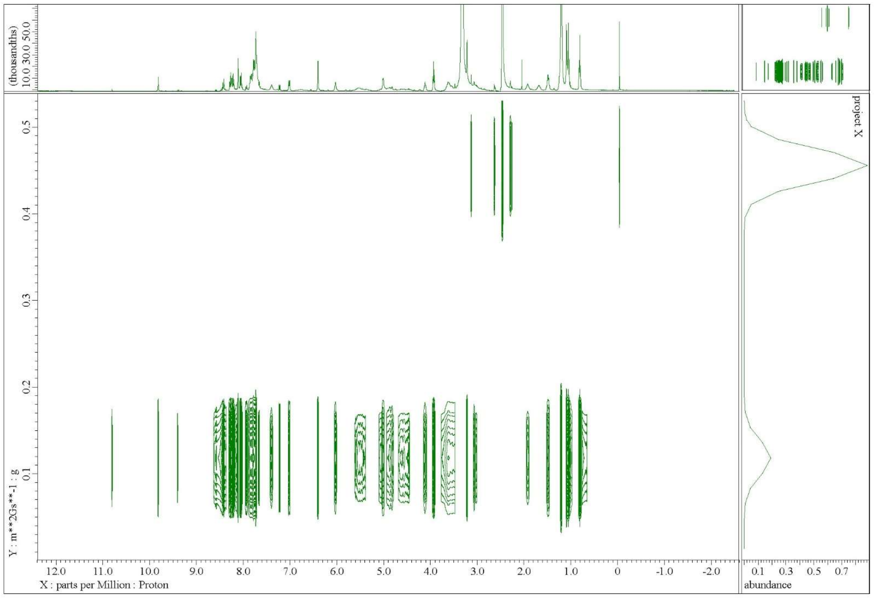

Figure S7. The DOSY NMR (400 MHz, DMSO-D$_6$, 25 °C) spectrum of 4-OI/TPP/RB@Zn-NH-pyr at a molar ratio of Zn-NH-pyr:4-OI:TPP:RB = 1:1:1:3.

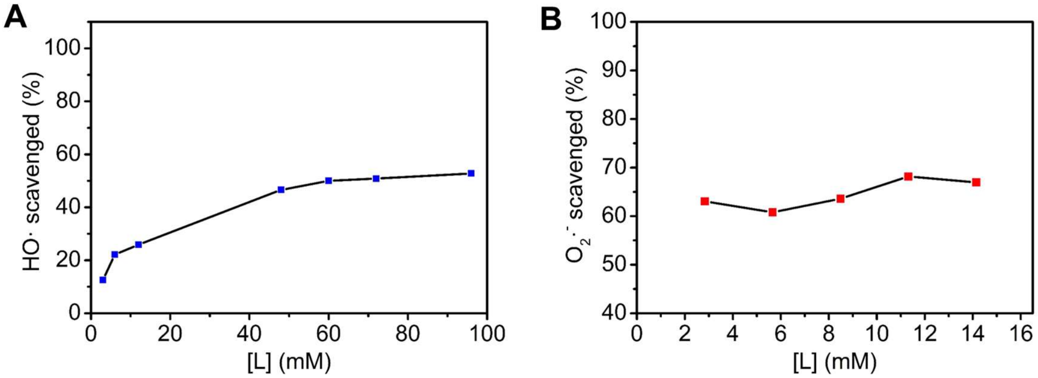

Figure S8. The plots of extracellular ROS scavenging of dicarboxylate ligand (L). (A) Hydroxyl radicals (HO•) scavenging activity determined at the condition of 3 mL of $FeSO_4$ (aqueous solution, 2.0 mM), 3 mL of H_2O_2 (aqueous solution, 2.0 mM), and 3 mL of SA in ethanol (2.0 mM) with 0.5 mL of various concentration of L in DMSO (3.0 – 96.0 mM). (B) Superoxide radicals ($O_2•^-$) scavenging capacity evaluated at the condition of 0.1 mL of riboflavin (0.2 mM), 0.1 mL of methionine (125 mM), and 0.1 mL of NBT (0.75 mM) in a PBS solution (pH = 7.4) with 0.1 mL of various concentration of L in DMSO (2.8 – 14.2 mM) followed by illuminatiing upon UV light for 1.5 min.

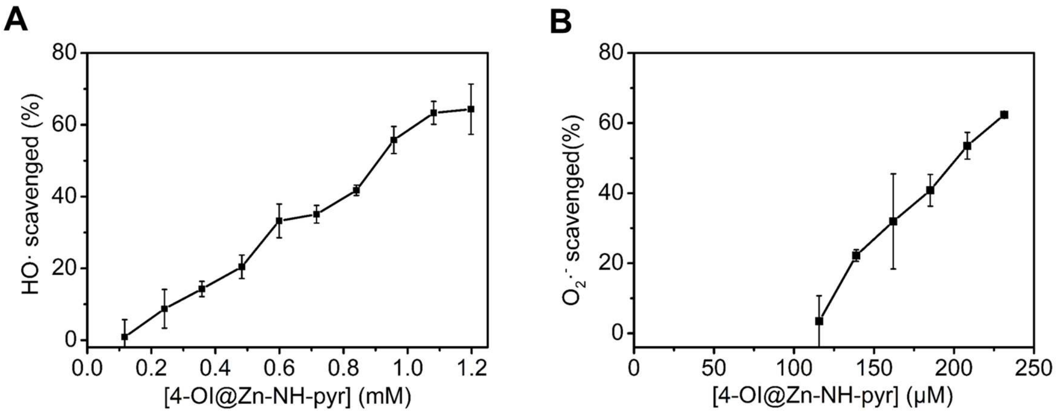

Figure S9. The plots of extracellular ROS scavenging of 4-OI@Zn-NH-pyr. (A) Hydroxyl radicals (HO•) scavenging activity determined at the condition of 3 mL of $FeSO_4$ (aqueous solution, 2.0 mM), 3 mL of H_2O_2 (aqueous solution, 2.0 mM), and 3 mL of SA in ethanol (2.0 mM) with 0.5 mL of various concentrations of 4-OI@Zn-NH-pyr in DMSO (0.12 – 1.2 mM) (mean ± SD, n = 3 independent samples). (B) Superoxide radicals (O_2•$^-$) scavenging capacity evaluated at the condition of 0.1 mL of riboflavin (0.2 mM), 0.1 mL of methionine (125 mM), and 0.1 mL of NBT (0.75 mM) in a PBS solution (pH = 7.4) with 0.1 mL of various concentrations of Zn-NH-pyr in DMSO (120 – 240 μM) followed by illuminating upon UV light for 1.5 min (mean ± SD, n = 3 independent samples).

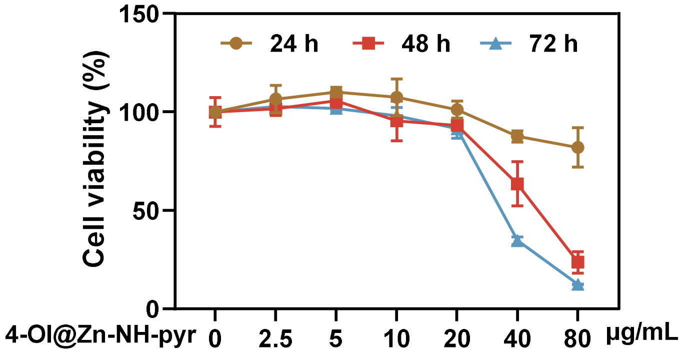

Figure S10. Cell viability of RAW 264.7 macrophages treated with various concentrations of 4-OI@Zn-NH-pyr for 24 , 48 and 72 h. Data are presented as the mean ±SD (n = 3 independent samples).

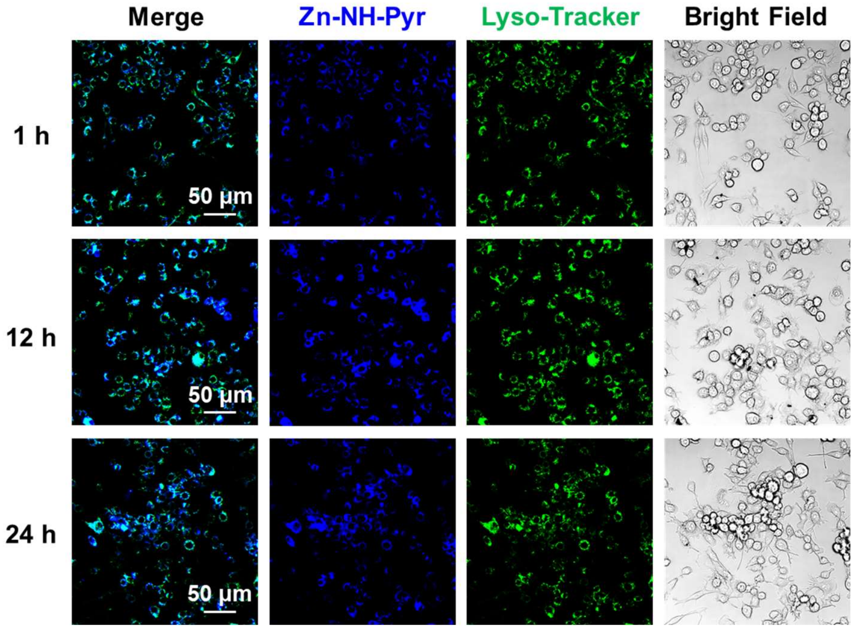

Figure S11. Confocal images of RAW 264.7 macrophages incubated with Zn-NH-pyr for 1 , 6 and 12 h. Lyso-Tracker was used for staining of endo/lysosomes.

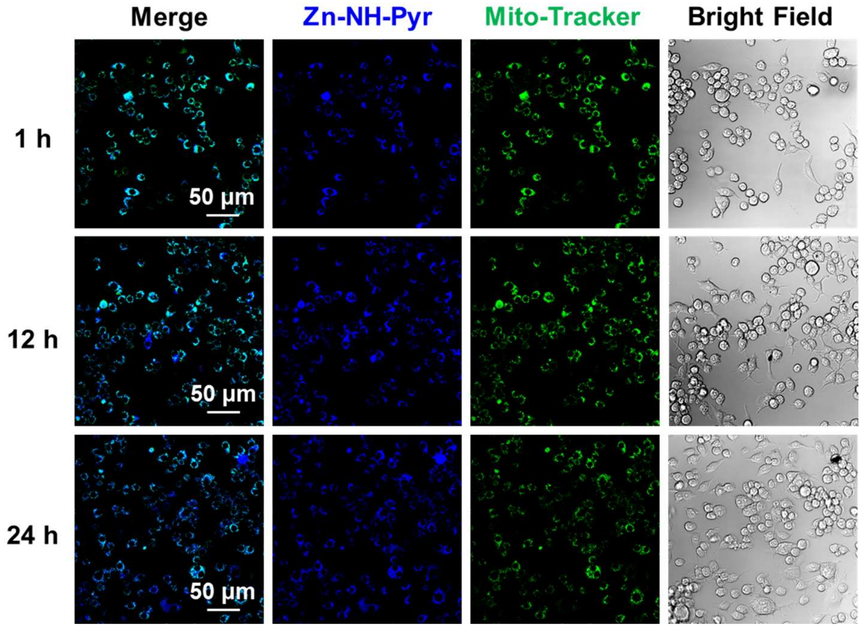

Figure S12. Confocal images of RAW 264.7 macrophages incubated with Zn-NH-pyr for 1, 6 and 12 h. Mito-Tracker was used for staining of Mitochondria.

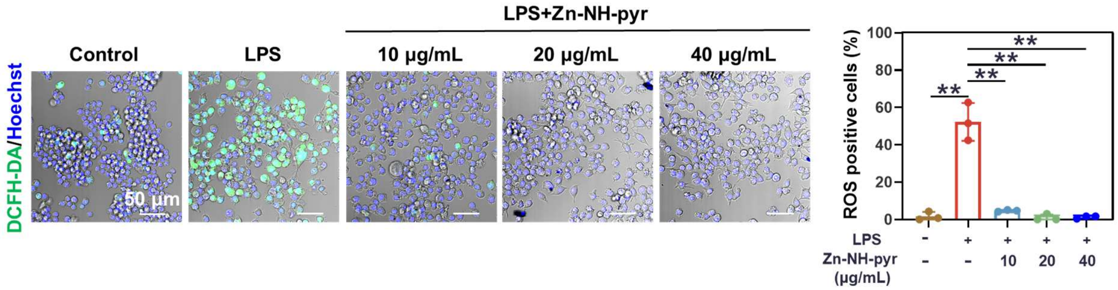

Figure S13. Confocal images of LPS-activated macrophages with various concentrations of Zn-NH-pyr, stained with DCFH-DA. Data are presented as the mean ±SD (n = 3 independent samples). Statistical significance was determined by one-way ANOVA with Tukey's multiple comparison test. $**P < 0.01$.

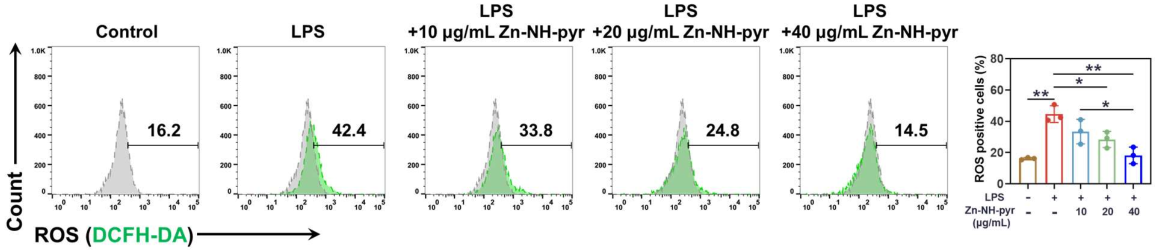

Figure S14. Flow cytometry of DCFH-DA-positive macrophages with various concentrations of Zn-NH-pyr. Data are presented as the mean ±SD (n = 3 independent samples). Statistical significance was determined by one-way ANOVA with Tukey's multiple comparison test. $*P < 0.05$; $**P < 0.01$.

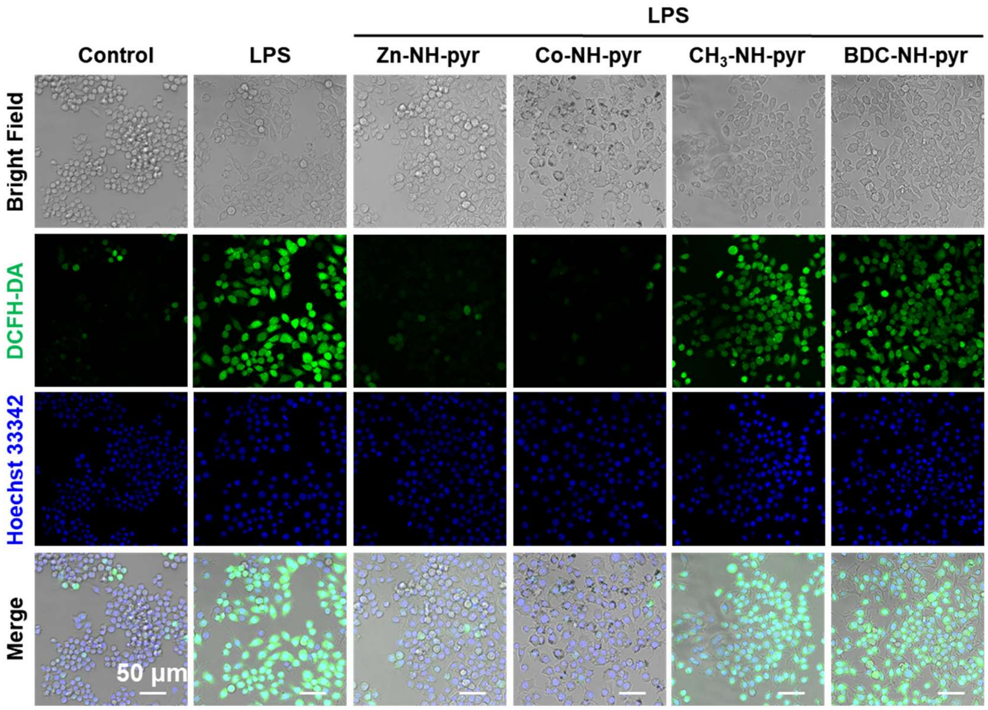

Figure S15. Confocal images of LPS-activated macrophages with various treatment groups, stained with DCFH-DA.

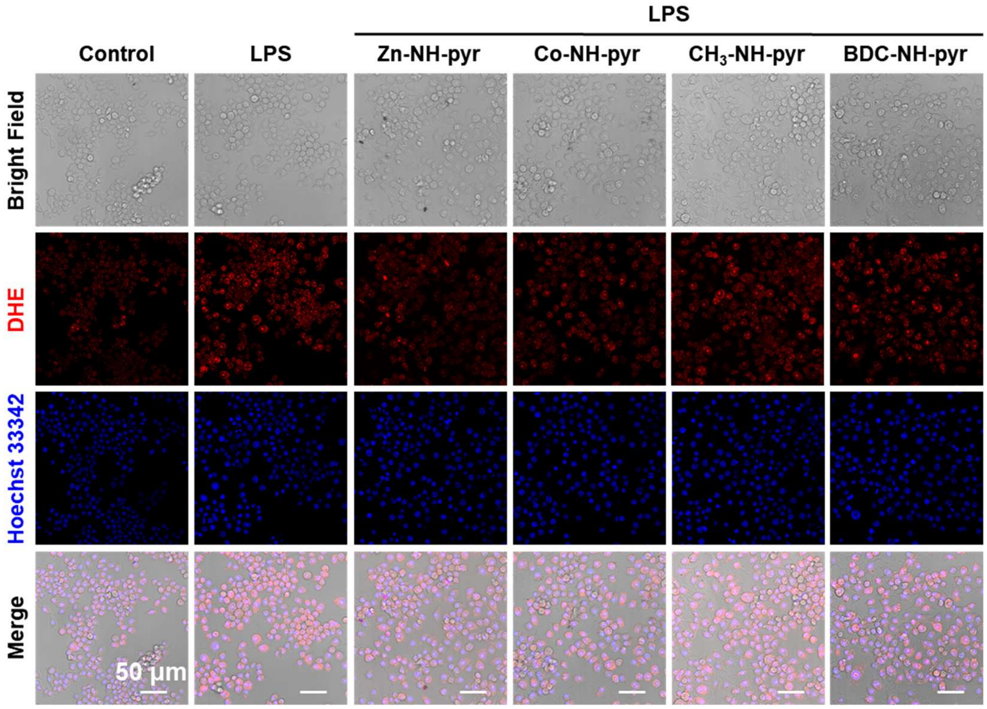

Figure S16. Confocal images of LPS-activated macrophages with various treatments, stained with DHE.

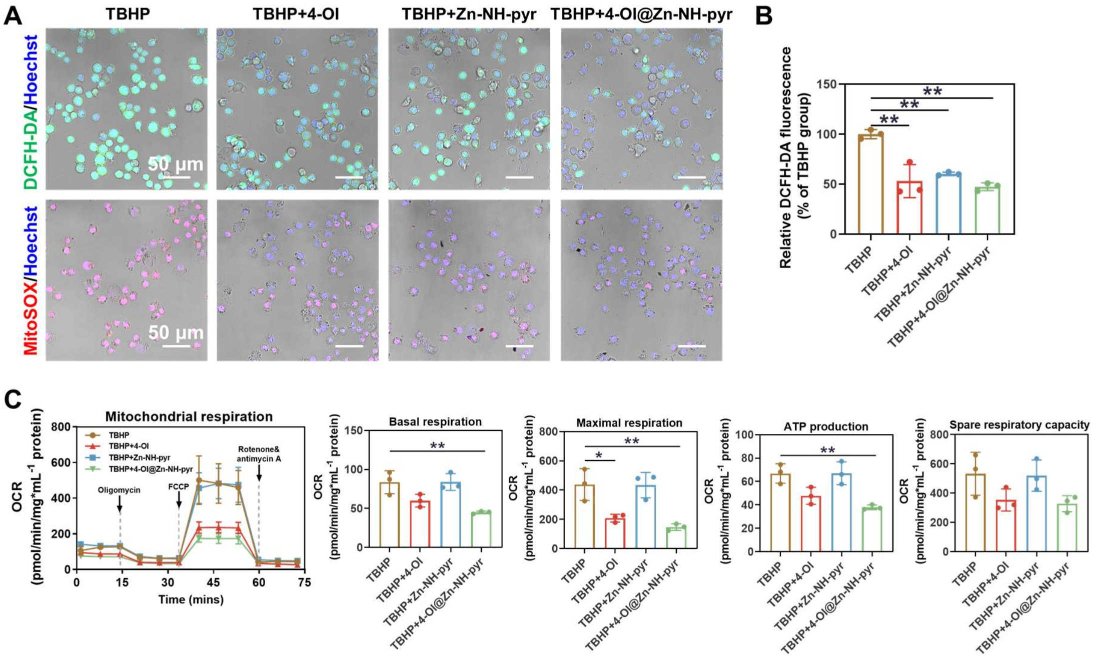

Figure S17. Effect of Zn-NH-pyr, 4-OI and 4-OI@Zn-NH-pyr on intracellular ROS and OCR of THP-1 macrophages in the presence of TBHP. (A) Confocal images of macrophages with various treatment groups stained with DCFH-DA or MitoSOX. (B) Percentage of DCFH-DA fluorescence intensity of various treatment groups relative to the Control group. (C) OCR of THP-1 macrophages with various treatment groups (mean ± SD, one-way ANOVA with Tukey's multiple comparison test, n = 3 independent samples). *$P < 0.05$; **$P < 0.01$.

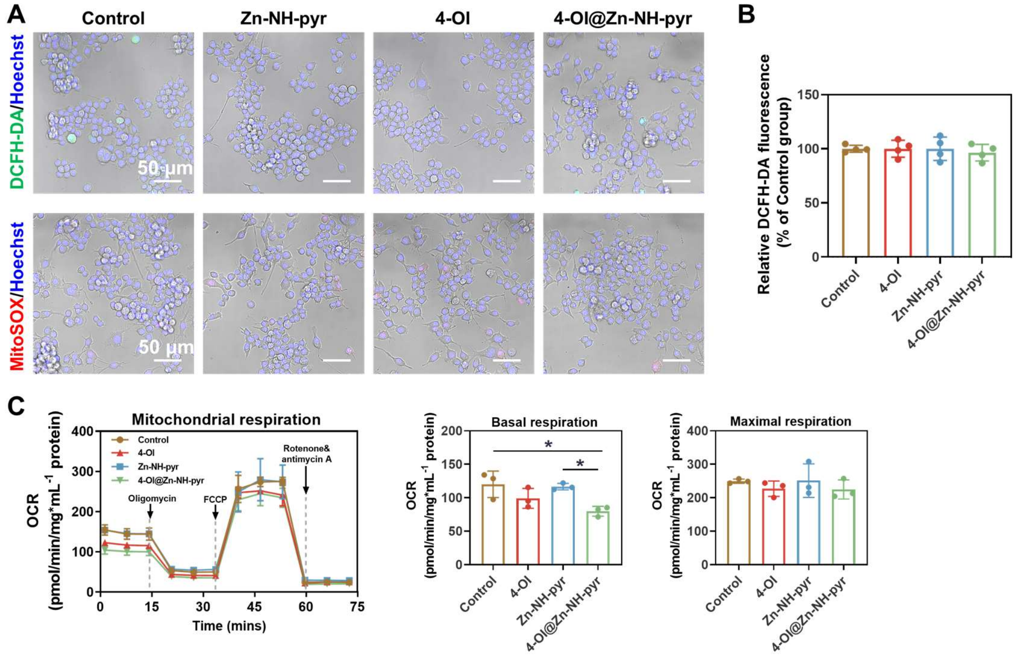

Figure S18. Effect of Zn-NH-pyr, 4-OI and 4-OI@Zn-NH-pyr on intracellular ROS of RAW 264.7 macrophages in basal condition. (A) Confocal images of macrophages with various treatment groups stained with DCFH-DA or MitoSOX. (B) Percentage of DCFH-DA fluorescence intensity of various treatment groups relative to the Control group. (C) OCR of RAW 264.7 macrophages with various treatment groups (mean ± SD, one-way ANOVA with Tukey's multiple comparison test, n = 3 independent samples). **P* < 0.05.

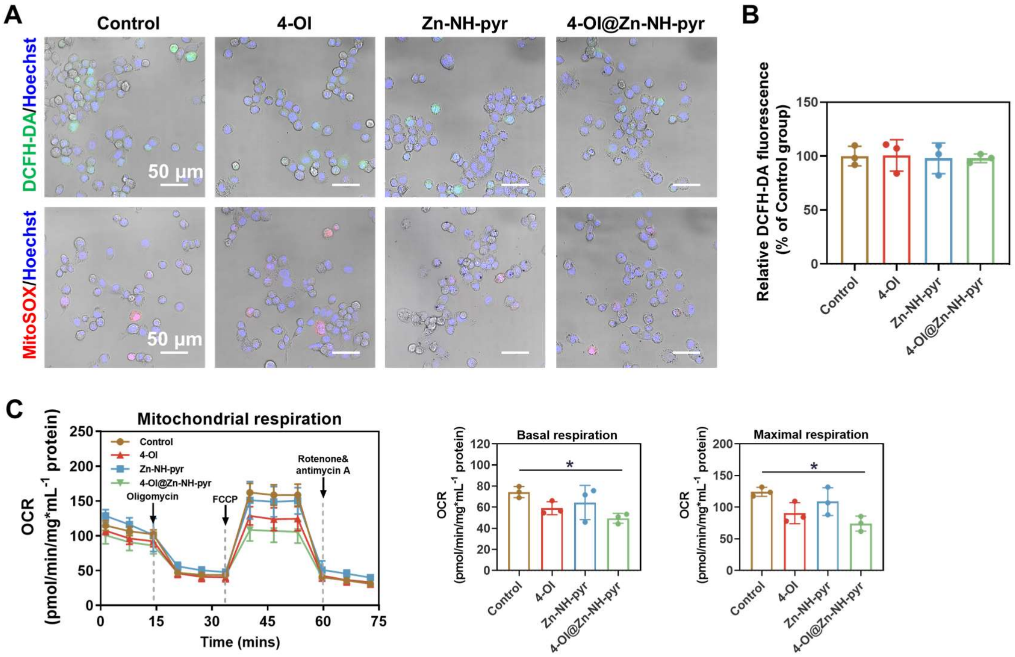

Figure S19. Effect of Zn-NH-pyr, 4-OI and 4-OI@Zn-NH-pyr on intracellular ROS and OCR of THP-1 macrophages in basal condition. (A) Confocal images of macrophages with various treatment groups stained with DCFH-DA or MitoSOX. (B) Percentage of DCFH-DA fluorescence intensity of various treatment groups relative to the Control group. (C) OCR of THP-1 macrophages with various treatment groups (mean ± SD, one-way ANOVA with Tukey's multiple comparison test, n = 3 independent samples). **P* < 0.05.

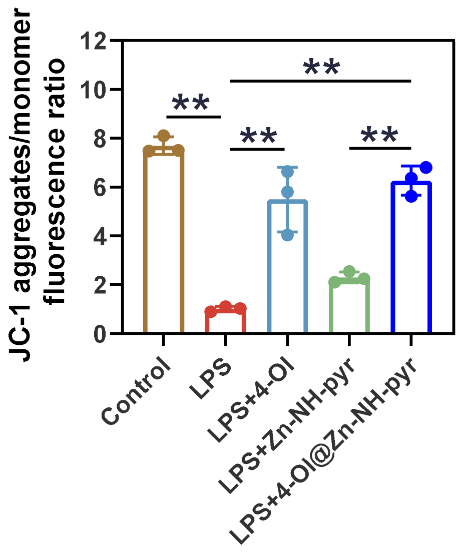

Figure S20. Quantitative analysis of the JC-1 aggregates/monomer fluorescence ratio (mean ± SD, one-way ANOVA with Tukey's multiple comparison test, n = 3 independent samples). $**P < 0.01$.

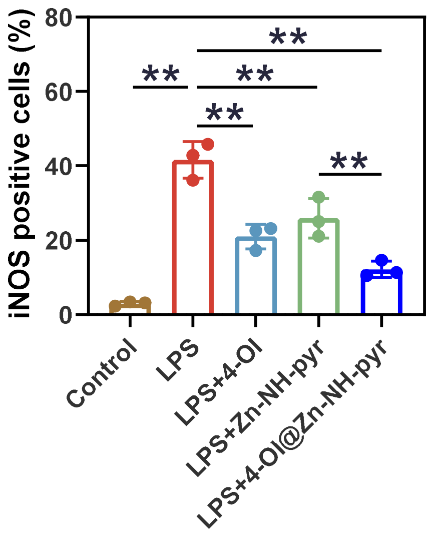

Figure S21. Quantitative analysis of the percentage of iNOS-positive cells (mean ± SD, one-way ANOVA with Tukey's multiple comparison test, n = 3 independent samples). $**P < 0.01$.

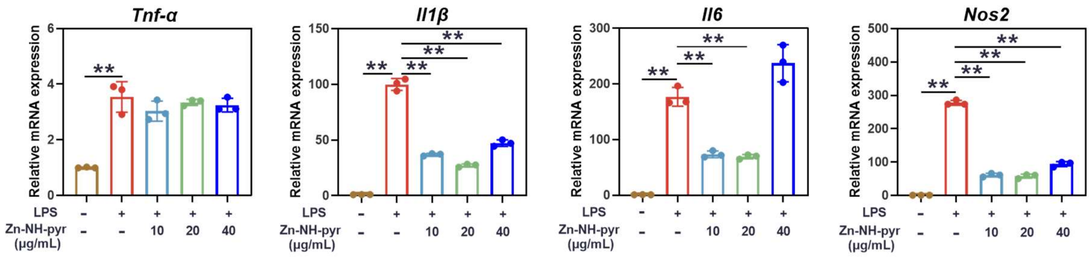

Figure S22. Expression of pro-inflammatory genes evaluated by RT-qPCR in LPS-activated macrophages with various concentrations of Zn-NH-pyr. Data are presented as the mean ±SD (n = 3 independent samples). Statistical significance was determined by one-way ANOVA with Tukey's multiple comparison test. ***P* < 0.01.

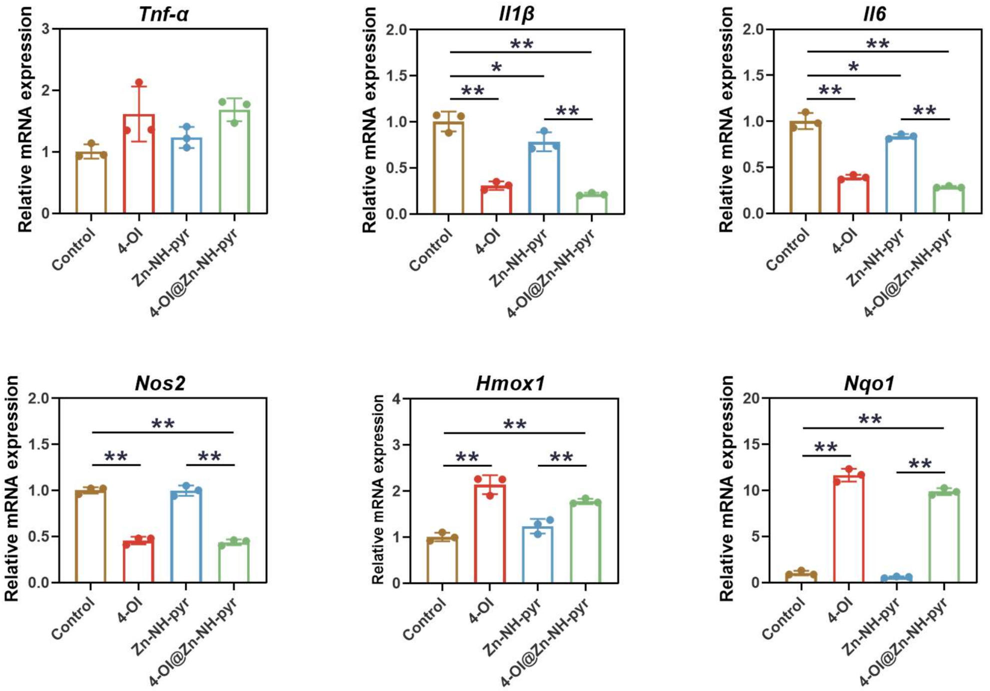

Figure S23. Expression of inflammatory-related genes evaluated by RT-qPCR in macrophages under basal conditions (mean ± SD, one-way ANOVA with Tukey's multiple comparison test, n = 3 independent samples). **P* < 0.05; ***P* < 0.01.

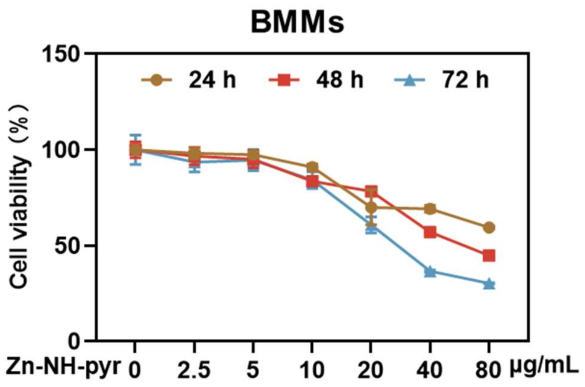

Figure S24. Cell viability of BMMs treated with various concentrations of Zn-NH-pyr for 24 , 48 and 72 h. Data are presented as the mean ±SD (n = 3 independent samples).

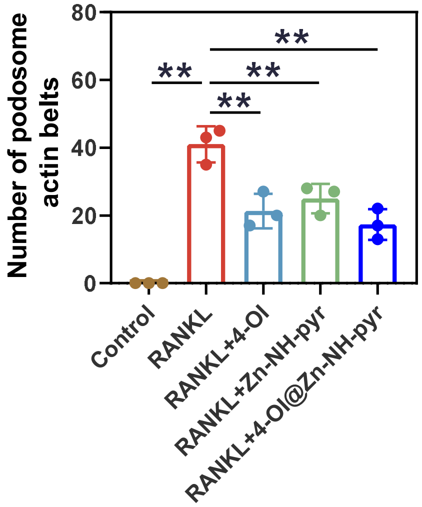

Figure S25. Quantitative analysis of the number of podosome actin belts in RANKL-stimulated BMMs with various treatment groups (mean ± SD, one-way ANOVA with Tukey's multiple comparison test, n = 3 independent samples). $^{**}P < 0.01$.

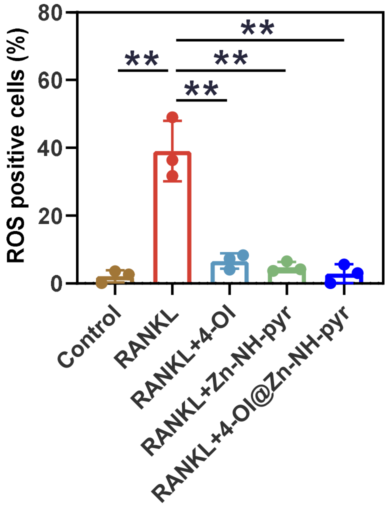

Figure S26. Quantitative analysis of RANKL-stimulated BMMs with various treatment groups stain with DCFH-DA (mean ± SD, one-way ANOVA with Tukey's multiple comparison test, n = 3 independent samples). $^{**}P < 0.01$.

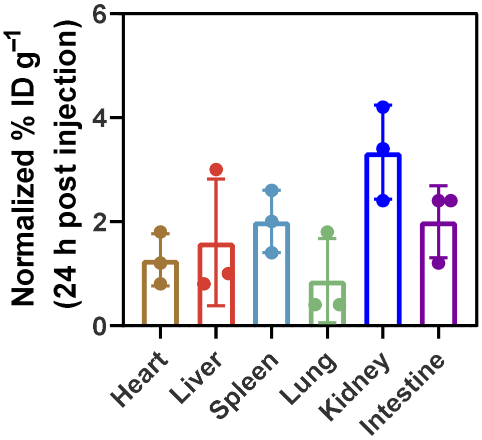

Figure S27. Biodistribution of Zn-NH-pyr in major organs at 24 h post injection (n = 3 independent animals).

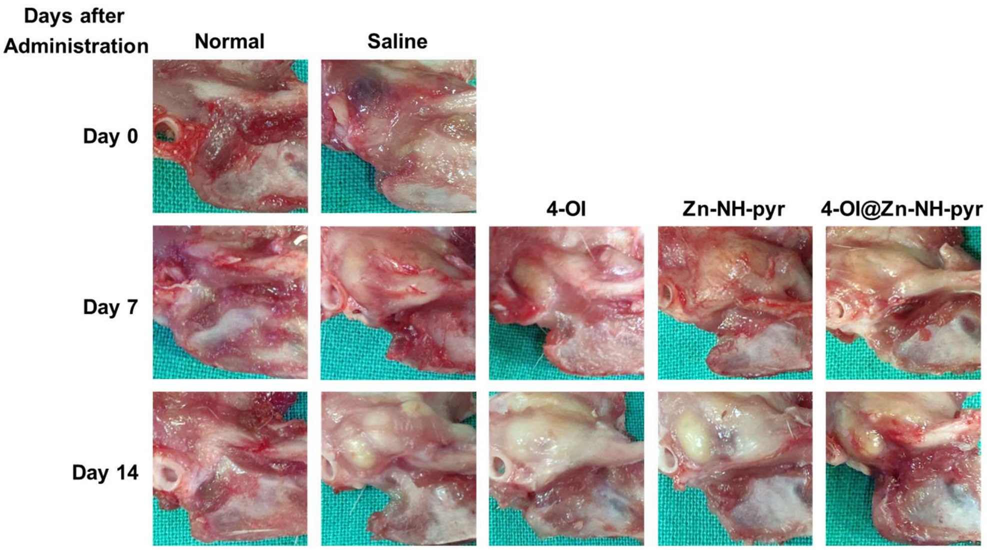

Figure S28. General histopathologic changes in the rat TMJ with various treatment groups (n = 3 independent samples).

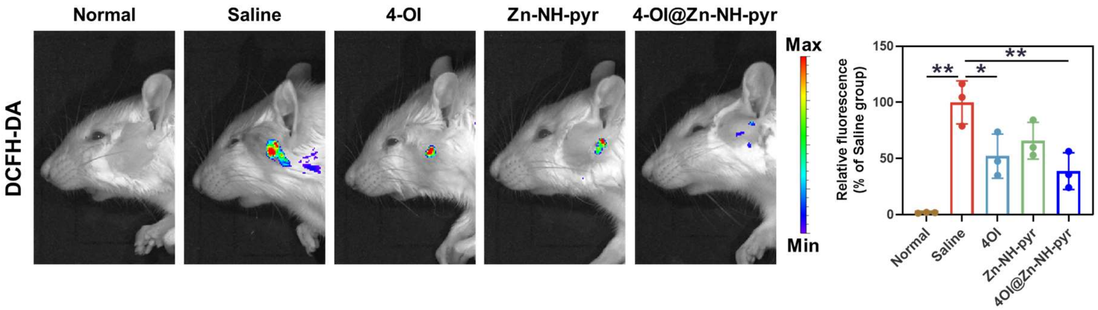

Figure S29. IVIS images of in vivo ROS level in rat TMJ with various treatment groups. Data are presented as the mean ±SD (n = 3 independent animals). Statistical significance was determined by one-way ANOVA with Tukey's multiple comparison test. *$P < 0.05$; **$P < 0.01$.

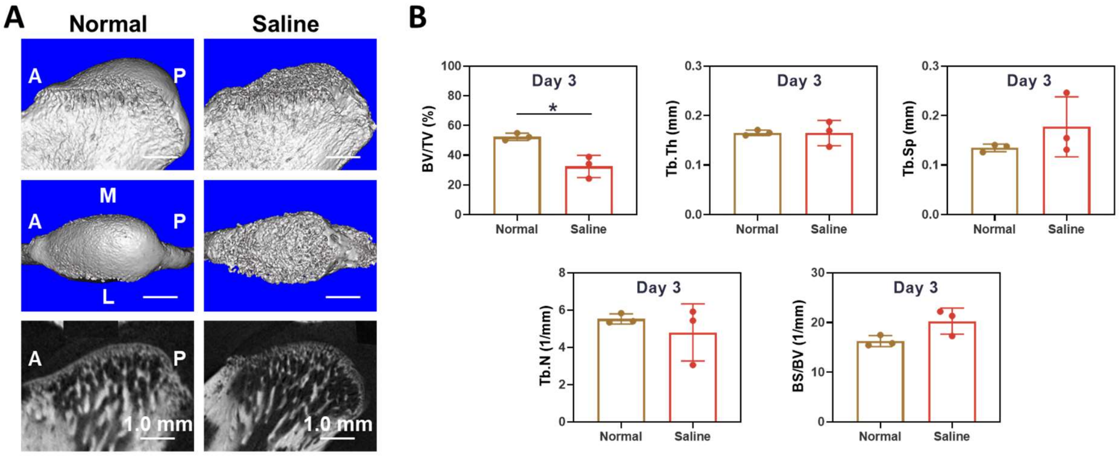

Figure S30. Micro-CT scanning and analysis of rat TMJ 3 days after immunization. (A) Representative images of micro-CT scanning of rat TMJ 3 days after immunization (n = 3 independent animals). (B) Quantitative micro-CT analysis of rat TMJ 3 days after immunization. Data are presented as the mean ±SD (n = 3 independent animals). Statistical significance was determined by one-way ANOVA with Tukey's multiple comparison test. *$P < 0.05$.

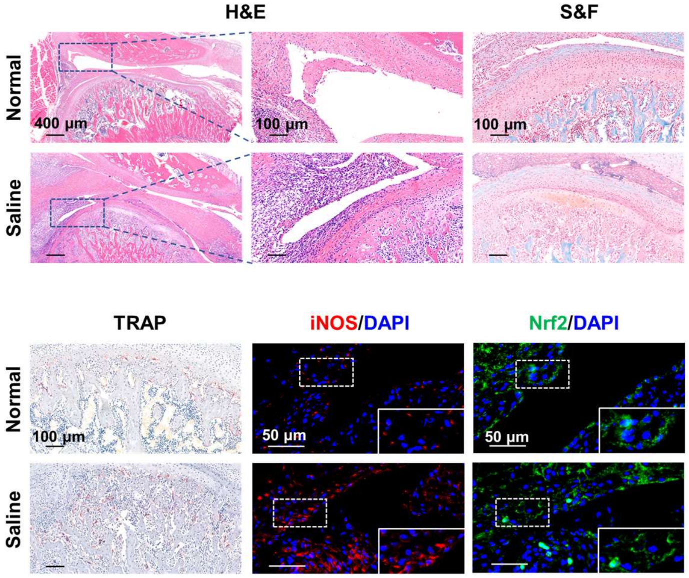

Figure S31. Representative images of H&E, S&F, TRAP and immunostaining of iNOS and Nrf2 3 days after immunization.

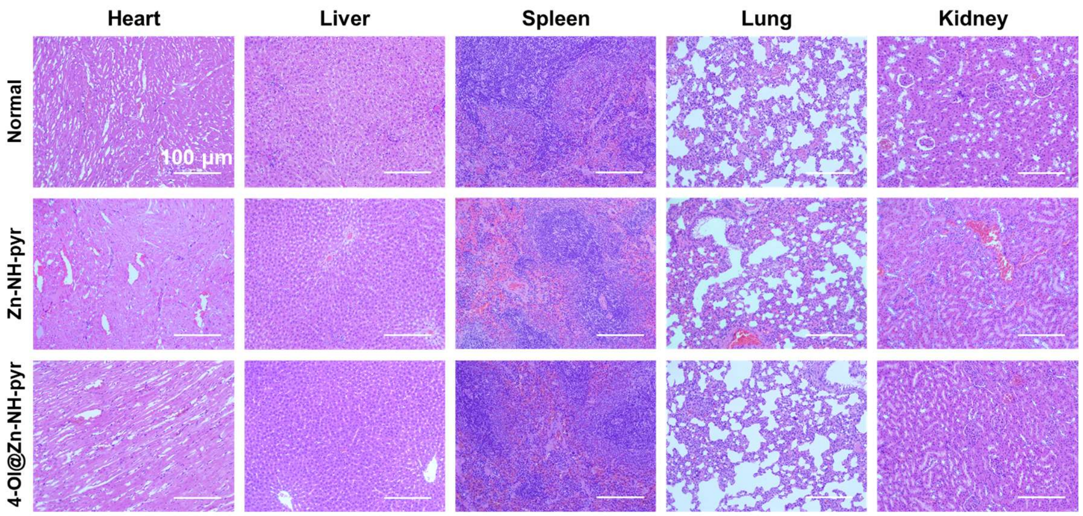

Figure S32. H&E staining images of heart, liver, spleen, lung, and kidney 14 days after treatment.

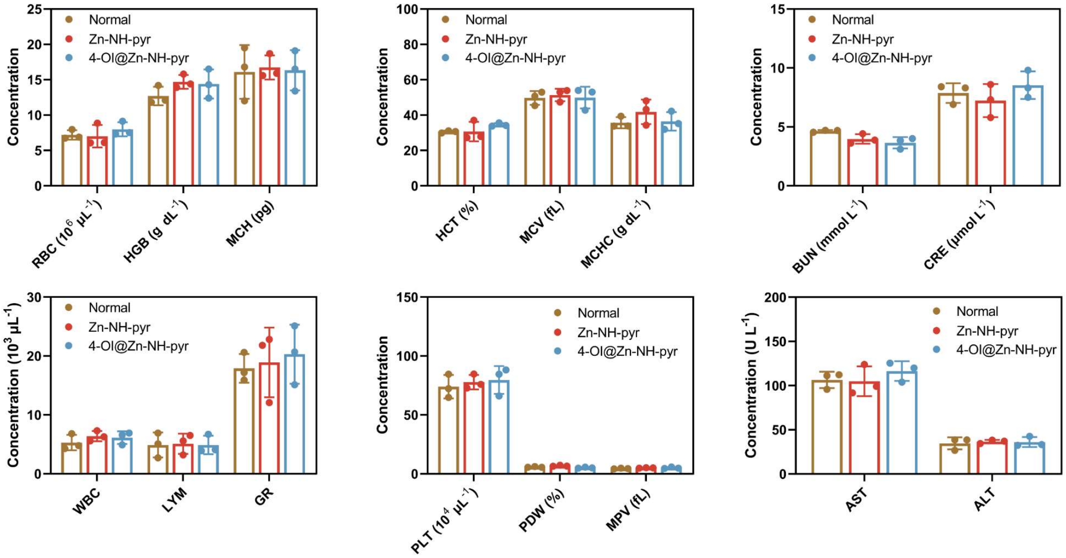

Figure S33. In vivo biosafety evaluation of Zn-NH-pyr and 4-OI@Zn-NH-pyr. Complete blood panel analysis and serum biochemistry analysis were performed 14 days after treatment. Data are presented as the mean ±SD (n = 3 independent animals).

Supplementary Tables

Table S1. Sequences of the RT-qPCR.

Gene	Forward	Reverse
Gapdh	ACCCAGAAGACTGTGGATGG	CACATTGGGGGTAGGAACAC
Tnf-α	GCCTCTTCTCATTCCTGCTTGTGG	GTGGTTTGTGAGTGTGAGGGTCT
Il1β	TCGCAGCAGCACATCAACAAGAG	AGGTCCACGGGAAAGACACAGG
Il6	CTTCTTGGGACTGATGCTGGTGAC	AGGTCTGTTGGGAGTGGTATCCT
Nos2	ACTCAGCCAAGCCCTCACCTAC	TCCAATCTCTGCCTATCCGTCTCG
Hmox1	GAGCAGAACCAGCCTGAACT	AAATCCTGGGGCATGCTGTC
Nqo1	GGTAGCGGCTCCATGTACTC	CGCAGGATGCCACTCTGAAT
Trap	CCATTGTTAGCCACATACGG	CACTCAGCACATAGCCCACA
Nfatc1	TGCTCCTCCTCCTGCTGCTC	GCAGAAGGTGGAGGTGCAGC
Ctr	TGCAGACAACTCTTGGTTGG	TCGGTTTCTTCTCCTCTGGA
Dcstamp	AAAACCCTTGGGCTGTTCTT	AATCATGGACGACTCCTTGG
Atp6v0d2	AAGCCTTTGTTTGACGCTGT	TTCGATGCCTCTGTGAGATG
Ctsk	CTTCCAATACGTGCAGCAGA	TCTTCAGGGCTTTCTCGTTC

References

1. Chen X, Chen X, Zhou Z, Mao Y, Wang Y, Ma Z, et al. Nirogacestat suppresses RANKL-Induced osteoclast formation in vitro and attenuates LPS-Induced bone resorption in vivo. Exp Cell Res. 2019; 382: 111470.
